# Synergistic effects of plant genotype and soil microbiome on growth in *Lotus japonicus*

**DOI:** 10.1093/femsec/fiae056

**Published:** 2024-04-27

**Authors:** Masaru Bamba, Turgut Yigit Akyol, Yusuke Azuma, Johan Quilbe, Stig Uggerhøj Andersen, Shusei Sato

**Affiliations:** Graduate School of Life Sciences, Tohoku University, 2-1-1 Katahira, Aoba, Sendai 980-8577, Japan; Department of Molecular Biology and Genetics, Aarhus University, DK-8000 Aarhus, Denmark; Graduate School of Life Sciences, Tohoku University, 2-1-1 Katahira, Aoba, Sendai 980-8577, Japan; Department of Molecular Biology and Genetics, Aarhus University, DK-8000 Aarhus, Denmark; Department of Molecular Biology and Genetics, Aarhus University, DK-8000 Aarhus, Denmark; Graduate School of Life Sciences, Tohoku University, 2-1-1 Katahira, Aoba, Sendai 980-8577, Japan

**Keywords:** 16S rRNA sequencing, cross-inoculation experiment, growth environment, *Lotus japonicus*, plant–microbiome interaction, root microbiome

## Abstract

The biological interactions between plants and their root microbiomes are essential for plant growth, and even though plant genotype (G), soil microbiome (M), and growth conditions (environment; E) are the core factors shaping root microbiome, their relationships remain unclear. In this study, we investigated the effects of G, M, and E and their interactions on the *Lotus* root microbiome and plant growth using an *in vitro* cross-inoculation approach, which reconstructed the interactions between nine *Lotus* accessions and four soil microbiomes under two different environmental conditions. Results suggested that a large proportion of the root microbiome composition is determined by M and E, while G-related (G, G × M, and G × E) effects were significant but small. In contrast, the interaction between G and M had a more pronounced effect on plant shoot growth than M alone. Our findings also indicated that most microbiome variations controlled by M have little effect on plant phenotypes, whereas G × M interactions have more significant effects. Plant genotype-dependent interactions with soil microbes warrant more attention to optimize crop yield and resilience.

## Teaser

Cross-inoculation experiments of 72 plant–microbiome–environment combinations showed that plant growth is more sensitive to plant genotype-dependent interactions with soil microbiomes than sole differences in microbiomes.

## Introduction

The interaction between the microbiome and plant roots is an important factor affecting plant growth. These interactions are pervasive and can have extensive effects on host plants, including disease resistance (Santhanam et al. 2015, Busby et al. 2016, Carrion et al. 2019), stress tolerance (de Vries et al. 2019, Liu et al. 2020), nutrient supply (Zhang et al. 2019), and overall plant health (Berendsen et al. 2012). Consequently, there has been increasing interest in determining how plant–microbiome interactions are established and maintained, as well as which effects the plant–root interactions depend on in order to establish sustainable agricultural systems and improve our understanding of ecosystems (Mauchline and Malone 2017).

In the complex ecosystem of plant–microbiome interactions, the root microbiome is shaped by an assemblage of factors, namely plant genotype (G), soil microbiome composition (M), and the environmental conditions under which the plant grows (E). Genetic differences between plants are one of the most studied factors affecting the root microbiome structure (Weinert et al. 2011, Bulgarelli et al. 2012, Lundberg et al. 2012, Peiffer et al. 2013, Walters et al. 2018, Brown et al. 2021). Phylogenetic distance between plants and root microbiome dissimilarity appears to be correlated at the interspecies, or higher, taxonomic levels (Bouffaud et al. 2014, Schlaeppi et al. 2014, Terrazas et al. 2020, Wang and Sugiyama 2020). In addition, the soil microbiome, being a vast microbial pool, substantially influences the root microbiome (Edwards et al. 2015). Soil factors, including soil type (Schreiter et al. 2014), nutrient content (Yeoh et al. 2016, Agri et al. 2022), and pH (Qi et al. 2018), regulate soil microbe diversity and offer a wide range of microbes that roots can use to form their microbiome. Macroenvironment, comprising of abiotic factors such as soil moisture (Bouskill et al. 2013, Naylor et al. 2017) and light conditions (Martin et al. 2018), also has significant effects on the root microbiome. These environmental conditions can affect both plant physiology and the activity and survival of soil microbes, thereby influencing root microbiome composition. Noting the interconnected influence of these factors, isolating and understanding their individual impact in experimental systems becomes increasingly critical.

Many studies have attempted to understand the individual effects of plant genotypes (G), soil microbiomes (M), and environmental conditions (E) on the root microbiome and their impact on plant growth (Lundberg et al. 2012, Yeoh et al. 2016, Naylor et al. 2017, Gallart et al. 2018, Azarbad et al. 2022). Nevertheless, two main obstacles interfere with our understanding of these complex relationships. First, isolating the M and E factors in field experiments is challenging because of the inherent complexities and interactions between the existing soil microbiome and environmental factors. Significant microbiome alterations have been reported in different soils and in soils exposed to different treatments (Rousk et al. 2010). Secondly, the scope of plant genotypes used in previous studies, as shown in tomato research (Oyserman et al. 2021), tends to be limited, which may restrict our understanding of the effects of plant genetic variation. Despite meaningful progress in understanding the individual effects of these factors, there still is a need to understand their combined and interactive effects. Numerous studies have documented interactions between individual microbes and plant genetic variation affecting plant growth (as reviewed by Bamba et al. 2018), suggesting a probable interaction between plant genetic variation and the root microbiome on plant growth. Yet, these interaction effects are not fully elucidated. Given these gaps, it is essential to use experimental systems that carefully reconstruct the effects of G, M, and E to better understand their influence on plant phenotypes.

Consequently, this study aimed to evaluate the impacts of G, M, and E, and their interactions on the root microbiome and plant growth. To disentangle these effects, we established an *in vitro* experimental system by combining nine genetically differentiated *Lotus* accessions, three soil microbiomes (and one microbiome-absent control), and two different environmental conditions. The soil microbiomes used in this study were extracted from two adjacent fields in northern Japan: one that had been subjected to salt treatment for 3 years and the other that had not. Furthermore, we included a combined community of both microbiomes and a noninoculated control for four inoculation groups. Environmental conditions were set to reflect those in which the soil microbiomes were collected, with or without salt treatment (adding 100 mM NaCl to the medium). In the present study, plants were grown under 72 combinations of these conditions, and the root microbiome and plant phenotypes were compared. The effects of G, M, and E, and their interactions on the root microbiome and plant growth were quantitatively assessed by examining the collected data.

## Materials and methods

### Cross-inoculation experiments

A cross-inoculation experiment was performed to quantify the effects of G, M, and E, and their interactions on plant phenotypes and root microbiomes. Nine *Lotus* accessions with four soil microbial inoculations (two microbiomes from the soil, one mixture, and a noninoculated control) were cultivated under two environmental conditions, resulting in 72 combinations.

Eight *Lotus japonicus* natural accessions (Gifu, MG11, MG20, MG46, MG56, MG63, MG67, and MG68) and one *Lotus burttii* (B-303) were used for the cross-inoculation experiment. Three strains (Gifu, MG20, and *L. burttii*) were selected based on their previous use as experimental lines (Kawaguchi et al. 2000, 2005). The other accessions were selected based on their genomic relationships and referred to as Group 1 (MG67 and MG68), Group 2 (MG56 and MG63), and Group 3 (MG11 and MG46) (Shah et al. 2020). Seeds of *Lotus* accessions were obtained from the National BioResource Project in Japan.

Soil microbiomes were obtained from soils collected in May 2020 from the Kashimadai fields of Tohoku University (38.46°N, 141.09°E) in northern Japan. Soil samples were obtained from two adjacent plots (Field No. 5 Control and Field No. 5 Salt Treated; F5C and F5S, respectively), where *L. japonicus* has been cultivated for the last 3 years. The salt treatment in F5S was conducted from 2017 to 2019 by irrigation with underground water containing salt at a concentration approximately equivalent to one-fourth of seawater concentration, which is about 0.8% (8 g/l) (Shah et al. 2020). F5C was simultaneously irrigated with regular water. A total of 5 kg of soil were collected from five sampling points far enough away from each other to avoid sampling bias. Prior to sample collection, at least 15 cm of soil were first removed to avoid sampling surface soil. The soils collected from each respective location were crushed as finely as possible using a hammer. The soil samples were separated into 250 g batches of each soil and crushed using a mixer and 250 ml of cold phosphate-buffered saline (PBS). The crushed soils were precipitated by centrifugation at 1000 × *g* for 10 min at 10°C, and the supernatants were collected. The precipitates were returned to the mixer and the process was repeated three times to extract the microbes in the clumps. The collected supernatant was filtered using Advantec 5A filter paper (particle size >7 µm; ash content <0.01%). The filtered solutions were centrifuged at 8000 × *g* for 20 min at 10°C, and the pelleted microbes (fungi and protists should be removed in the filtration and precipitation processes) were collected and diluted with 250 ml of PBS to obtain a 1 ml per g soil microbial community extract (250 ml of extract from 250 g of soil). Microbial community extracts from F5C, F5S, and a 1:1 mixture (MIX) were used for the cross-inoculation experiments. As the difference in OD600 values between the F5C and F5S extracts was less than 2%, their concentrations were not adjusted.

To establish differences in the growth environment corresponding to the soils used for the extraction of microbiomes, two types of media (salt-treated and control) were used for plant cultivation. Initially, two 20 l tanks of B&D medium (Broughton and Dilworth 1971) were prepared, each enhanced with 1 mM KNO3 to restrict symbiotic interactions with nitrogen-fixing nodule bacteria, thereby ensuring plant growth independence from these bacteria. Subsequently, NaCl was added to one tank to achieve a concentration of 100 mM. The medium from each tank was then distributed into four 5 l tanks, totaling eight tanks, which were autoclaved to sterilize. Six of these tanks were supplemented with one of three microbial communities—F5C, MIX, and F5S—at a 1% (v/v) concentration, with two tanks designated for each SALT and non-SALT condition. The medium from these eight tanks was then allocated into 19 pots filled with vermiculite, culminating in 152 pots. These pots were utilized for transplanting the plants in the ensuing experiments.

Partly scrubbed *Lotus* seeds were sterilized by immersion in 2% sodium hypochlorite for three min and rinsed three times with sterile MilliQ water. After overnight immersion, the swollen seeds were sown on 1% agar plates, incubated in the dark for 3 days at 25°C, and then grown at the same temperature under 16/8 light/dark conditions for 24 h. Rooted plants were transplanted into pots with lids, filled with 300 ml of sterilized vermiculite and 250 ml of media, and grown at 25°C under the same light conditions for 4 weeks. Growth pots were closed with lids to prevent cross-contamination. Two pots were used for each plant–microbiome–environment combination. A total of 144 pots (nine plant accessions × four inoculants × two conditions) were simultaneously grown in a growth chamber. The 144 pots were randomly assigned to 10 groups (14 or 15 pots per group) and placed on trays. To prevent uneven placement of the pots, pot positions in the tray and the position of the trays in the growth chamber were rotated every week. Furthermore, once every 2 weeks, the groups of pots were changed randomly. To avoid heterogeneous lighting conditions, a growth chamber with lights on the top was used and photon flux densities ranging from ~140–150 µmol/m^2^/s were maintained. Six plants were cultivated per pot. The detailed growth system was shown in Fig. S1 (Supporting Information).

The whole plant bodies were harvested, all individuals were imaged using a high-resolution scanner, and their roots and root nodules were separated. Individuals whose first leaves could not be observed were considered as dead plants were not included in observational data, since they did not possess sufficient roots to detect their root microbiomes. The shoot length (SL), number of leaves (NOL), number of branches (NOB), and root length (RL) were measured from the scanned data as plant phenotypes. The roots were washed with sterilized distilled water, frozen in liquid nitrogen, and preserved at −80°C until DNA extraction.

### DNA extraction for MiSeq sequencing

A total of six to seven living individuals were selected from each combination of G, M, and E (72 combinations, three individuals of noninoculant samples) to identify root microbiomes; when sufficient individuals were collected from two pots, the selection of individuals from each pot was kept equal as possible. Prior to DNA extraction, each root sample was cut into ~2 cm pieces and randomly collected into sterilized tubes. Genomic DNA of each root sample was extracted using the Qiagen MagAttract 96 DNA Plant Core Kit (QIAGEN Inc., Valencia, CA, USA) according to the manufacturer’s instructions. In addition, three DNA samples were extracted from each of the soil extracts (F5C and F5S; Day 0) used in the experiments. Six DNA samples for each combination were extracted from the soil collected from nonplant pots (F5C, MIX, and F5S in both saline and nonsaline environments), which had been kept stationary while the plants were growing. Each soil microbiome genomic DNA sample was extracted using the FastDNA Spin Kit for Soil (MP Biomedicals, Illkirch, France).

Pair-end library preparation for MiSeq sequencing was conducted using the two-step tailed PCR method described by Illumina (San Diego, CA, USA). The following primer pairs were used to amplify partial sequences of the 16S rRNA gene:

V5F_MAUI_799 (forward): 5′-TCGTCGGCAGCGTCAGATGTGTA TAAGAGACAGNNNHNNNWNNNHAACMGGATTAGATACCCKG-3′

V7R_1192 (reverse): 5′-GTCTCGTGGGCTCGGAGATGTGTATAA GAGACAGACGTCATCCCCACCTTCC-3′

The 3′ end to 18 bases and 19 bases of each primer (forward and reverse, respectively) were 16S rRNA universal sequences (Chelius and Triplett 2001). The 19–30 base from the 3′ end of the forward primer is a unique molecular identifier (Fields et al. 2021). The other regions were the Illumina Overhang adapter sequences. Second-round PCR was performed using primer pairs with 16 unique indices: D501–D508 and A501–A508 (forward) and D701–D712 and A701–A712 (reverse) (Illumina).

The DNA concentrations of the purified PCR products were adjusted and pooled into two different tubes, as the MiSeq run was performed in two separate runs. The 16 × 24 indexing system used could not accommodate six samples per condition, necessitating the division to analyze our sample set. The samples used in the experiments are listed in Table S1  (Supporting Information). The paired-end libraries were mixed with 3% PhiX DNA spike-in control and used for sequencing on the MiSeq platform using Illumina MiSeq v.3 Reagent kit for 2 × 300 bp PE. All the sequence files obtained in this study have been deposited in the DDBJ database (DRR511003–DRR511426).

### Data analysis for microbiome

Quality control was performed for the sequenced reads and paired-end read assembly using PEAR v0.9.6 (Zhang et al. 2014). The low-quality tails in each read were trimmed using a Phred score of 20 as the threshold, and trimmed reads with lengths less than 200 bp were discarded. Paired-end reads with an overlap of more than 10 bp and a total length of more than 300 bp were combined. The UMI, primer, and target sequence regions of each read were identified based on sequence length. UMIs were counted without duplication, and the abundance of each sequence was determined based on the number of UMIs. This process can inhibit multiple counts of amplified products derived from the same molecule, thus reducing the PCR amplification bias. Sequences with an average number of UMIs greater than or equal to 0.05 for all samples were chosen and used in the following analyses. UMI identification and counting were performed using Python 3 software (https://github.com/mbamba2093/Maui-seq_for_microbiome).

A BLAST search (Camacho et al. 2009) was conducted for each sequence using a database containing the RDP11 bacterial 16S rRNA sequences (Cole et al. 2014) and *L. japonicus* Gifu genome v1.2 (Kamal et al. 2020) to assign the sequences to microbial taxa. Part of the classification rank of RDP11 that was out of alignment was manually corrected. All taxa, except for species-level assignment (taxonomic levels were as follows; phylum, order, class, family, and genus), were compared with the List of Prokaryotic names with Standing in Nomenclature (Parte et al. 2020) to prevent misclassification, and those that did not match were marked as “NotAssigned.” For the BLAST results, multiple sequences had the highest match rate, and the sequence with the exact genus name or earliest RDP ID was selected. The sequences that showed the highest match rate in the BLAST search of the *L. japonicus* Gifu genome but not in the RDP11 database were plant-derived and were excluded. All remaining sequences were analyzed as amplicon sequence variants (ASVs). Based on the BLAST top hit results, each ASV sequence was categorized into taxonomic groups (domain, phylum, class, order, family, and genus), and sequences with the same top hit were classified as OTU.

### Microbial community analysis

Community analyses were performed to evaluate the effects of combinations of G, M and E, and their interactions with the plant root microbiome. The following analyses were performed using data aggregated from sequences with the same OTU, genus, family, and order.

Prior to the analysis, to reduce biases due to differences in sampling depth, the community data was subsampled based on the rarefaction curve using the *rarefy* function implemented in vegan R (Oksanen et al. 2020). The sample with the lowest community coverage was identified based on the slope at the endpoint of the rarefaction curve and the number of reads was adjusted to match this slope for all samples (Chao and Jost 2012). The rarefied community data was converted into frequency data.

The effects of G, M, and E on diversity were calculated using a generalized linear model (GLM), excluding noninoculant and soil samples. In the GLM, α-diversity was the response variable and the effects of G, M, and E and their interactions were explanatory variables. Gamma distribution was chosen as the error distribution and the log-link function for the model. Statistical significance was evaluated using the *F*-test. A separate GLM was constructed for the soil samples. The first model was used to evaluate differences between F5C and F5S at the time of inoculation. The second model was used to evaluate the effects of M and E and their interactions 28 days after inoculation. For significant variables in the *F*-test (*P* < .05), the Tukey–Kramer test was conducted to compare α-diversity among all groups. The vegan package (Oksanen et al. 2020) were used to calculate diversities and the *glm, Anova*, and *glht* functions in R 3.6.1 (R core team, 2019) were used to estimate the effects of G, M, E, and interactions.

To distinguish root microbiome structures, β-diversities among samples were calculated using the Morisita–Horn index (Horn 1966) since the index is likely to be resistant to undersampling bias (Wolda 1981) and the data from short-read sequences could be ambiguous with other phylogeny-based β-diversities. To visualize the similarity of microbiomes, a nonmetric multidimensional scaling (nMDS) analysis was conducted using the *metaMDS* function in vegan R (Oksanen et al. 2020) with 100 random parameters. In the nMDS analysis, four different assessments were conducted: one incorporating all samples, one excluding noninoculated samples, one excluding both noninoculated and soil samples, and another utilizing only soil samples. PERMANOVA with the *adonis* function in vegan, R (Oksanen et al. 2020) was used with 99 999 permutations to evaluate which factors shape the microbiome structure. PERMANOVA was conducted exclusively with root microbiome samples, excluding noninoculated samples. To estimate the effects of variation within *L. japonicus* species on the root microbiome, β-diversity analyses were performed using these data, excluding *L. burttii*. These analyses were also conducted for each microbiome–environment combination to clarify the effects of the host genotype in different combinations.

In addition, the following permutation analyses were performed to deal with potential confounding factors caused by each pot, since the individual plants in the same pot shared a unique environment. One of the pots from each combination was selected to exclude pot bias and G, M, and E and their interaction effects on α- and β-diversity were evaluated using the GLM model and PERMANOVA for 1000 permutations.

Furthermore, the correlation between host genomes and root microbiome differences in each microbiome–environment combination was also investigated using the Mantel test implemented in the ape package in R (Paradis and Schliep 2019). Genetic distances of *L. japonicus* genomes were calculated using identical-by-state kinships based on the population genome information reported by Shah et al. (2020). The pairwise similarity distances of the microbiomes were calculated using the Morisita–Horn index, which was calculated by averaging the microbiomes of each host.

The effects of G, M, E, and their interactions on the frequency of individual bacteria (OTU-level classification) were estimated using a nonparametric regression model called the generalized smoothing model (GSM). Bacterial OTUs observed in more than six individual plants were selected for analysis to exclude excessive results from bacteria with minor distributions. In the model, each bacterial frequency was the response variable and the effects of G, M, and E and their interactions were explanatory variables. Nominal spline smoothing was chosen as the smoothing function for all explanatory variables, with default settings for the other parameters. Statistical significance was evaluated using the *F*-test. Model fitting and *F*-tests were implemented in the npreg package in R3.6.1 (Heiwig 2022). Fisher’s exact test was used to evaluate whether the significantly affected strains were distributed disproportionately in specific families using R3.6.1.

### Data analysis for plant phenotypes

Heat maps were first generated using host-standardized phenotypic values, whose mean values for each host genotype were set to zero to visualize the variation in phenotypes. Correlations among phenotypes were estimated using Pearson’s product–moment correlation. To detect the effects of G, M, and E on the correlations among phenotypes, separate correlation tests were performed for each G, M, and E group. Heatmaps were illustrated using the *heatmap.2* program implemented in *gplots* in R3.6.1 (R Core Team 2019). Correlation analyses were performed using the function implemented in ggpairs of R3.6.1 (R Core Team 2019).

To analyze the effects of G, M, and E and their interactions on plant phenotypes (we focused on only SL and RL because all phenotypes were correlated with each other), a GLM was used instead of analysis of variance (ANOVA), because the distribution of phenotypic values deviated significantly from a normal distribution (Shapiro–Wilk test, *P*-value < .05, for all phenotypes). In the GLM, each phenotype was the response variable and the effects of G, M, and E and their interactions were explanatory variables. Gamma distribution was chosen as the error distribution and log link function for all phenotypes because the distribution did not deviate from the expected distribution. We calculated the type II sums of squares for each variable, evaluated their statistical significance using *F*-tests, and estimated each variable’s effect size (η^2^). In addition, the Tukey–Kramer test was performed to compare plant phenotypes among the G, M, and E groups. These analyses of variance were performed using the *Anova* function implemented in the *car* library (Fox and Weisberg 2019) and the *etaSquared* function implemented in the *lsr* library in R.3.6.1 (R Core Team, 2019). The Tukey–Kramer test was performed using the *glht* function implemented in the multcomp library in R.3.6.1 (Hothorn et al. 2008). The same analysis was performed using a dataset that excluded noninoculated individuals to evaluate the effects of differences in the inoculation community.

In addition, the following statistical analyses were performed to deal with potential confounding factors caused by each pot, since the individual plants in the same pot shared a unique environment. The interclass correlation coefficients (ICC: variance between pots/all variance) of pots for each combination of inoculation tests (72 G × M × E combinations) were evaluated using the *glmer* function in R3.6.1. The ICCs were calculated for two plant phenotypes, SL and RL, owing to the low variance in the other phenotypes. Even if there was bias due to the combination of pot effects, a multilevel analysis containing pot information as a random effect was unsuitable since the pot variables completely masked the combination information. One of the pots from each combination was randomly selected to exclude pot bias and G, M, and E and their interaction effect were then evaluated using the GLM model for 1000 permutations.

As both plant phenotypes and microbiomes depend on the effects of G, M, and E and their interactions, the extent to which root microbiome structures explain the variance in plant SL was calculated. The variance component was calculated using the following equation:


\begin{eqnarray*}
{\mathrm{Y\ }} = {\mathrm{\ \mu \ }} + {\mathrm{\ \varepsilon }},
\end{eqnarray*}

where Y is a SL vector standardized for each host accession, *ε* is an error term and *µ* is the similarity matrix of the root microbiome based on 1 - the Morisita–Horn similarity index matrix and the identical matrix used in the community analysis. The *emma* function in the R pipeline was used to calculate the variance component u (Kang et al. 2008).

All the R scripts used in this study are available at (https://github.com/mbamba2093/Plant-genotype-Root-microbiome-synergy).

## Results

A cross-inoculation experiment was performed using nine *Lotus* accessions (G) and three microbiome inoculants with one noninoculant control (M) under two environmental conditions (E), resulting in 72 combinations. 749 individuals (6–12 individuals per combination) were collected (Table S1, Supporting Information). Although 12 plants were cultivated for each combination, ~9% of these plants did not survive. On the roots of collected individuals, a few nodules observed (average 0.12 nodules on the root of one *Lotus* individual, except for noninoculant).

### Plant root microbiomes and effects of G, M, and E

The root microbiomes of 382 plants were investigated. Using MiSeq sequencing, 45 031 087 reads were obtained, preprocessed, and allocated to each individual (ranging from 13 878 to 198 897 per individual). All quality-filtered reads were used to count the UMI. In addition, 68 023 unique sequences with an average number of UMIs greater than or equal to 0.05, were used for the BLAST search. As a result of the BLAST search, 62 836 sequences consisting of 16 785 973 reads were derived from the bacterial 16S rRNA genes. Bacterial sequences were assigned to 5891 different bacterial OTUs (sequences assigned the same BLAST top hit were aggregated as an OTU-level taxon), 277 genera, 85 families, 41 orders, 22 classes, and 11 phyla (Supplementary materials). The largest proportion of the microbiome was Proteobacteria (root microbiome, 85%; soil microbiome at extraction, 61%; and soil microbiome at 28 d, 79%), followed by Bacteroidetes and Firmicutes, with these three phyla accounting for ~90% of the community (Fig. S2, Supporting Information). Meanwhile, Actinobacteria were observed in the soil microbiome (8.8% at extraction and 4.1% at 28 days), but rarely in the root microbiome (0.03%). Furthermore, our analysis detected few *Mesorhizobium* bacteria (occupying ~0.1% of the microbiome), which are common rhizobial symbionts of *L. japonicus* (Bamba et al. 2019).

**Figure 1.**
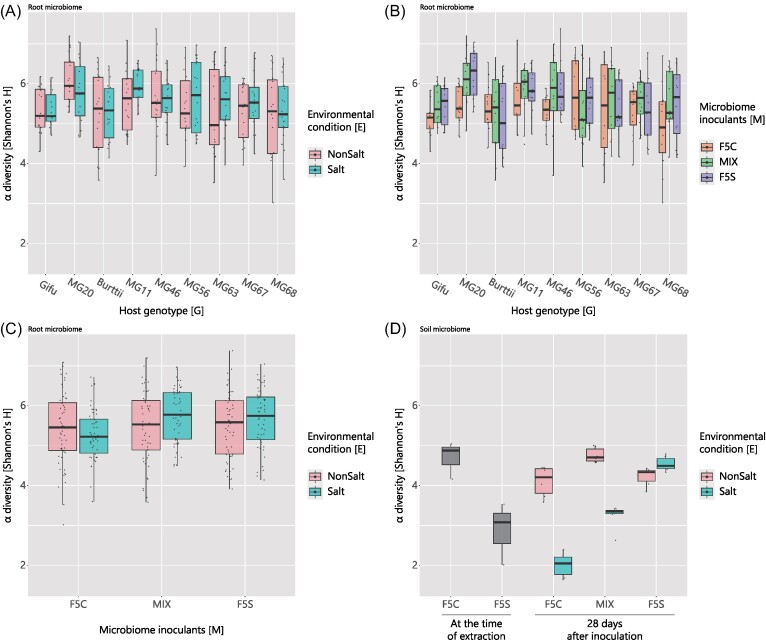
α-diversities of root and soil microbiomes. α-diversity based on the Shannon index with an OTU-level taxonomic assignment. (A), (B), and (C) Comparison of α-diversity of a root microbiome between combinations of two groups; combinations of host genotypes (G) and environmental conditions (E), that of (G) and microbiome inoculants (M), and that of (E) and (M), respectively. (D) Comparisons of α-diversity of a soil microbiome. The gray boxplots indicated the diversity at the time of extraction from soils and colored plots indicated the soil microbiome after 28 days from inoculation.

Prior to the diversity analysis, coverage-based rarefaction was performed and the lowest slopes at the end of the rarefaction curves were 0.1665, 0.0275, 0.0016, and 0.0005 for the ASVs, OTU, genus, and family levels, respectively (Fig. S3, Supporting Information). The α-diversities of the *Lotus* root microbiome were calculated using the Shannon index (Shannon and Weaver 1949) based on rarefied composition data without noninoculant root samples. The α-diversity at the microbiome OTU level ranged from 1.643 to 7.376 (Fig. 1). The α-diversity of the soil microbiomes was lower than that of the root microbiomes, and the diversity of the F5S inoculants was lower than that of F5C at the time of soil extraction (Fig. 1). At 28 days, the α-diversity of the soil microbiome did not differ between F5S and F5C in nonsalt environments (Tukey test; *P* = .9658), and the diversity was higher in the MIX-inoculated soils (MIX—F5C: *P* = .0031 and MIX—F5S: *P* = .0240). However, the diversities of the F5C- and MIX-inoculated soils were much lower in the salt environment than in the nonsalt environment (Tukey test; both *P* < .0001), and the diversity of the F5S-inoculated soils was slightly higher in the salt environment than in the nonsalt environment (*P* = .3791). In the F5C inoculated soils, 73% of OTUs were less frequent in salt environments than in nonsalt environments. This result is in line with the expectations of the experimental design, that microbiomes from salt-stressed environments do not decrease in diversity when exposed to nonsalt environments, whereas microbiomes from nonsalt environments are stressed in salt environments.

The same pattern for the α diversity of the root microbiome as for the soil microbiome was observed, but the effect was limited. A significant effect of G and C on the α-diversity was detected, whereas their interactions and the environment had no effect (*P* < .05; Table S2, Supporting Information). The Tukey–Kramer test indicated that the root microbiomes of MG20 were more diverse than those of Gifu and MG68 and that the microbiomes with MIX were more diverse than those with F5C inoculants (*P* < .05; Table S3, Supporting Information). The α-diversities at other taxonomic levels are listed in Table S1 (Supporting Information). On the other hand, only 20% of the microbes that were less frequent in saline environments in F5C-inoculated soils were also less abundant in the plant root microbiome grown in F5C-inoculated soils.

**Figure 2.**
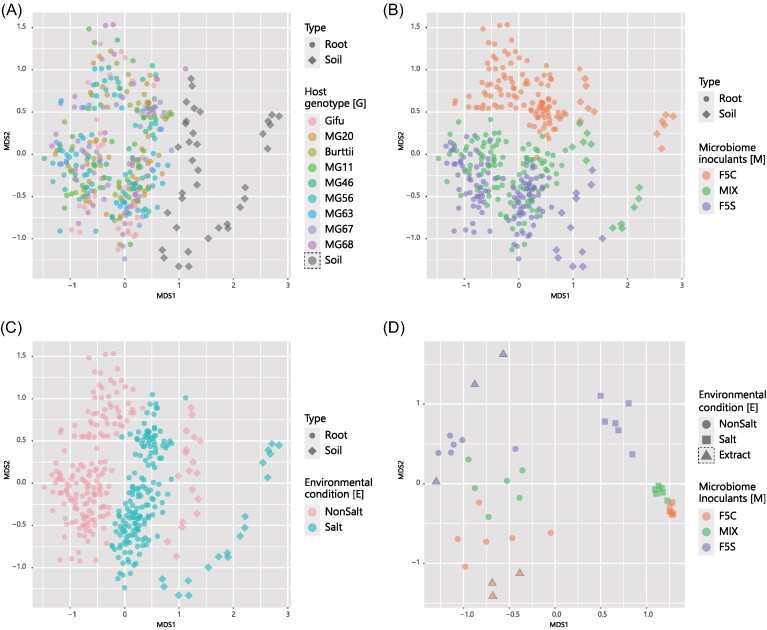
Microbiome structures based on β-diversity. nMDS for *Lotus* root and soil microbiome dissimilarity (Morisita–Horn index) is shown. (A), (B), and (C) Colors represent different plant genotypes, microbiome inoculants, and environmental conditions, and shapes represent the sample types. (D) Soil microbiome structures. Shapes represent environmental conditions in the experiments, and triangle shapes indicated the microbiomes at the time of extraction. Colors represent the microbiome inoculants, or original soils for the samples at the extraction.

The community structures of the root microbiomes were characterized based on the β**-**diversity (Morisita–Horn index). The nMDS analysis showed no overlap between the noninoculated samples and soil or root microbiomes (Fig. S4, Supporting Information). This apparent difference suggests that the microbiome detected in the noninoculated samples was either not removed by seed coat sterilization or was derived from contaminating bacteria during the experiment. The same data analysis without the noninoculated samples confirmed the root and soil microbiome community structures (Fig. 2). At the time of soil extraction (day 0 samples), a clear difference was observed between F5C and F5S microbiomes, indicating differences in the microbiomes between the two soils used in the experiment (Fig. 2D). A total of 4 weeks after inoculation, the nonsalt environment showed no apparent difference from the 0 days microbiomes, however, in the salt environment, the microbiomes deviated from the 0 days community, and the degree of deviation was greater for MIX and F5C than for F5S (Fig. 2D).

In the root microbiomes, the nMDS analysis showed an apparent difference between environments (E) and among inoculants (C), whereas the differences between hosts (G) were unclear (Fig. 2A–C). This pattern was observed across all taxonomic levels, although differences in the C effect were attenuated for family level classifications (Fig. S5, Supporting Information). PERMANOVA analysis indicated that G, M, and E, and their interactions significantly affected the root microbiome structure (*P* < .05; Table 1; Table S4, Supporting Information). At the OTU level microbiome, the effects of C and E were the largest, explaining about 22% of the variance. The others (G, G × M, G × E, M × E, and G × M × E) were around 4%, and 35% of the variance was residual. This result was comparable to that of the community structure of the root microbiomes of *L. japonicus* (Table S5, Supporting Information), indicating that the root microbiome of *L. burttii* accessions did not deviate from that of *L. japonicus*. Evaluating the G effect in conditions where the combination of M and E was fixed showed that the differences in G could explain 25%–40% of the variation in microbiome composition (Table S6, Supporting Information). In addition, differences in the root microbiome among host plants did not correlate with the genetic distances between host accessions (*P* > .05; Fig. S6 and Table S7, Supporting Information).

**Table 1.** Permanova results for variation in root microbiomes.

	DF^a^	SS^b^	F value^c^	*R* ^2^	*P*-value	
G	8	4.1482	4.2358	0.0438	1.00E-04	***
M	2	20.4385	83.4795	0.2160	1.00E-04	***
E	1	20.7195	169.2544	0.2189	1.00E-04	***
G × M	16	3.6435	1.8602	0.0385	1.00E-04	***
G × E	8	4.0255	4.1105	0.0425	1.00E-04	***
M × E	2	4.0812	16.6692	0.0431	1.00E-04	***
G × M × E	16	4.1637	2.1258	0.0440	1.00E-04	***
Residuals	273	33.4196		0.3531		
Total	326	94.6396		1		

a Degree of freedom.

b Sums of squares.

c Pseudo-*F* value in permutation.

To identify which bacterial OTUs were affected by G, M, and E; that would be masked by the β diversity analysis, these effects were evaluated using a nonparametric regression model, the GSM, in which the response variable was bacterial frequency. Of the 5510 OTUs (OTU-level classification that was observed in more than six plant individuals), 4712 were significantly affected by the G, M, and E variables and their interactions (Table S8, Supporting Information). The G variable had a significant effect on 218 of these strains; however, 2741 and 3318 strains were affected by M and E, respectively. Details of the overlap of each effect are shown in Fig. S7 (Supporting Information) as a Venn diagram. The strains affected by G, M, and E were shared by G vs. M (99 OTUs), G vs. E (122 OTUs), and M vs. E (1558 OTUs) (Fig. S7A, Supporting Information). Variables containing G (G, G × M, G × E, and G × M × E) significantly affected 269 OTUs (Fig. S7B, Supporting Information). Variables containing M and E (M, E, M × E, and G × M × E) significantly affected 4643 OTUs (Fig. S7C, Supporting Information). Three bacterial families, Bacillaceae, Flavobacteriaceae, and Methylophilaceae, were observed to be significantly sensitive by variables containing G (Bacillaceae: G, Flavobacteriaceae: G × M × E, and Methylophilaceae: G × M and G × M × E. Fisher’s exact test FDR-*P* < .05; Table S9, Supporting Information). These families accounted for 0.07%, 2.16%, and 1.57% of the microbiomes, respectively, ranking 22nd, 8th, and 10th out of 85 families, respectively.

### Plant phenotype and effects of G, C, and E

**Figure 3.**
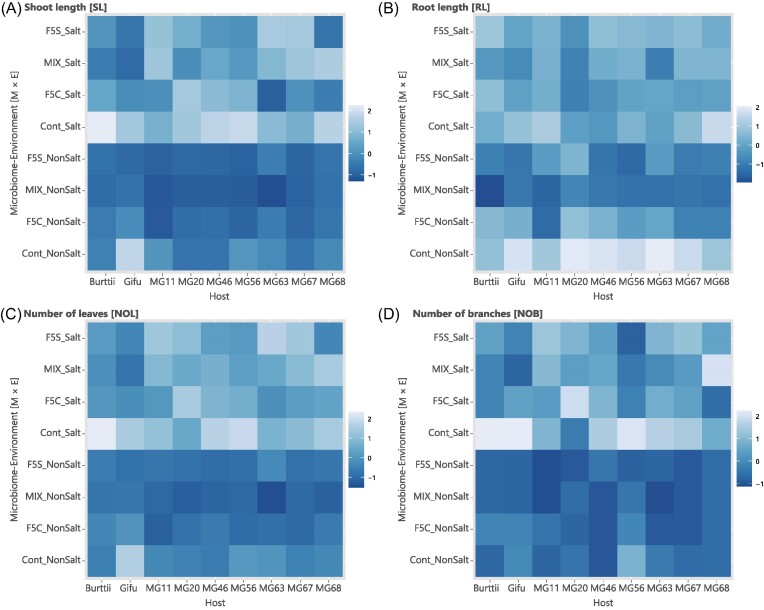
Plant phenotypic variation in the cross-inoculation experiments. Heatmaps of the four plant phenotypes: (A) SL, (B) RL, (C) NOL, and (D) NOB. Each cell color indicates standardized phenotypic values for each plant genotype.

Four phenotypes were obtained (SL, RL, NOL, and NOB) from 749 individuals (Fig. 3). All phenotypic traits were positively correlated (Pearson’s product–moment correlation: *P* < .001; Fig. S8, Supporting Information). All combinations of phenotypic traits, except those between RL and NOB, were significantly correlated in groups G, M, and E (Pearson’s product–moment correlation: *P* < .05; Figs S8–S10, Supporting Information). Because all four phenotypes were correlated, SL and RL were used in the following analyses as representatives of shoot and root phenotypes, respectively. The phenotypic distributions in all combinations were shown in Fig. S11 (Supporting Information).

In the cross-inoculation experiment, significant effects of G, M, and E and their interactions on SL and RL were detected using the GLM, except for M × E on SL (*F*-test *P* < .05; Table S10 and Fig. S12, Supporting Information). The most prominent effects on SL and RL were observed for G. The second largest effect on SL was on E, whereas that on RL was on M (Table S10, Supporting Information). The coefficients of salt addition as an E factor were positive for SL and RL, and the Tukey–Kramer test indicated significant differences between the salt and nonsalt environments (*P* < .001; Table S11, Supporting Information). It should be noted that the *Lotus* genus has different sensitivities to salt in soils between closely related species and within species, with MG20 and *L. burttii* being less inhibited by salt and Gifu being more inhibited. These results were consistent with those reported by Melchiorre et al. (2009). The C coefficients for F5C, MIX, and F5S in the GLM were mostly negative, except for F5C in RL. The Tukey–Kramer test showed significant differences between noninoculated and inoculated conditions (*P* < .001; Table S11, Supporting Information). This result indicated that inoculation of the microbiomes obtained from the Tohoku fields had adverse effects on plant growth.

**Figure 4.**
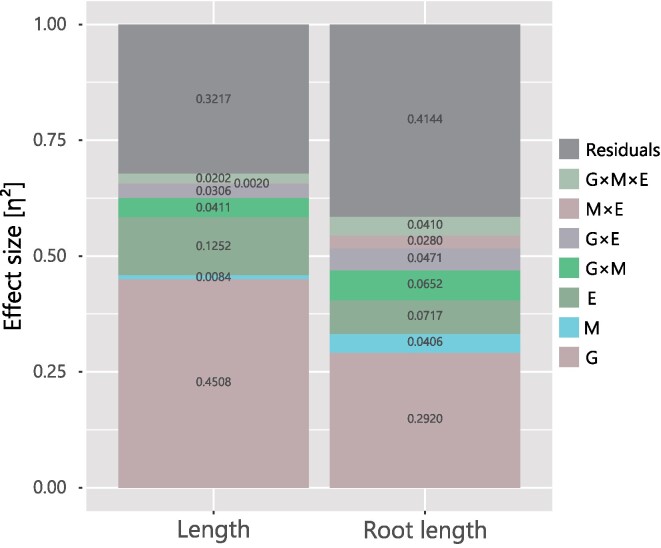
Effect sizes of G, M, E, and their interactions on plant phenotypes. The portion of each color and number on the bar chart represent the effect size η^2^ of each variable in the GLM without noninoculant data.

The GLM without noninoculant data showed that all G, M, and E cases and their interactions significantly affected SL and RL, except for M × E in SL (Table 2; Fig. S13, Supporting Information). The largest effect size among these models was G. The second largest effects on SL and RL were E and G × M, respectively. The M variables showed that the differences in the inoculant microbiomes in this model had a less significant effect on plant phenotypes (Fig. 4). The η^2^ of variables M for SL was 0.008, less than “small” by Cohen 1998s guideline (small: 0.01 <, moderate: 0.01 < 0.06, and Large: 0.06 <); meanwhile, that for RL was 0.047, which is moderate. The η^2^ of variable G × M on SL and RL were 0.041 and 0.062, which are assigned to “moderate” and "large,” respectively. These η^2^ values of G × M variables were larger than those of M variables for SL and RL, indicating that G × M variables were more significant than M.

**Table 2.** GLM for plant phenotypes in the cross-inoculation experiment without noninoculant data.

		DF^a^	SS^b^	*F*-value	η^2^	*P*-value	
SL^c^	G	8	142.7912	89.5144	0.4508	3.53E-92	***
	M	2	2.6831	6.7279	0.0085	1.31E-03	**
	E	1	39.6495	198.8470	0.1252	2.24E-38	***
	G × M	16	13.0035	4.0759	0.0411	2.21E-07	***
	G × E	8	9.6834	6.0704	0.0306	1.78E-07	***
	M × E	2	0.6252	1.5678	0.0020	2.10E-01	
	G × M × E	16	6.4003	2.0061	0.0202	1.14E-02	*
	Residuals	511	101.8919		0.3217		
							
RL^d^	G	8	55.8274	45.0118	0.2920	1.34E-54	***
	M	2	7.7633	25.0371	0.0406	4.23E-11	***
	E	1	13.7093	88.4267	0.0717	1.77E-19	***
	G × M	16	12.4628	5.0242	0.0652	9.96E-10	***
	G × E	8	9.0001	7.2564	0.0471	3.85E-09	***
	M × E	2	5.3483	17.2485	0.0280	5.64E-08	***
	G × M × E	16	7.8349	3.1585	0.0410	3.50E-05	***
	Residuals	511	79.2232		0.4144		
							

a Degree of freedom.

b Sums of squares.

c Shoot length.

d Root length.

In this study, the potential confounding factors derived from each pot could have caused an overestimation of all effects because pot differences masked all G × M × E combinations. First, the extent to which the variation in plant phenotypes could be explained by the differences in pots for each G × M × E combination was calculated. On average, 15% and 8% of the variance in SL and RL, respectively, was derived between pot replicates, indicating that variation existed between them. To assess if variations among pot replicates could lead to an overestimation of the effects, we conducted an analysis where one pot from each treatment combination was randomly selected and evaluate the impacts of G, M, E, and their interactions on plant phenotypes. Although the G × M × E effects could not be distinguished from the pot effects during the permutations, other effects could be estimated by considering the variability derived from the pot effects. From the permutation analysis results, variance was observed for all effects, whereas the distribution of each effect was comparable to that of the full dataset (Fig. S14, Supporting Information). For SL and RL, the effects of G were the largest. The second largest variables were E and G × M for SL and RL, respectively. A comparison of the effects of G × M and M on permutations showed that the number of trials with G × M > M was 95.1% in SL and 100% in RL, suggesting that the plant phenotypes were more sensitive to the interaction between G and M than M alone.

As both plant phenotypes and microbiomes depend on the effects of G, M, and E and their interactions, we attempted to integrate the variation in SL and root microbiome structure with variance component analysis. SL values standardized by the G factor were used to calculate SL variation because this factor explained a large amount of SL and little of the root microbiome structure. Variation in root microbiome structure was calculated based on the Morisita–Horn similarity index matrix, which is identical to the matrix used in community analysis. In this analysis, 35% of the variance in SL could be explained by the similarity in root microbiome structures. This result indicates that identifying which microbes could affect plant growth was difficult, even though many types of microbes in the soil microbiome would have favorable or adverse effects on plant phenotypes.

## Discussion

In the present study, we found that the synergistic effects of plant genotype and soil microbiome have larger impact on plant growth but different impacts on root microbiomes via 72 G × M × E combinations of *in vitro* inoculation experiments. The initial differences observed between the inoculant microbiomes at the point of collection from the soil allowed us to conduct an experiment that disentangled the effects of G, M, and E, and we could evaluate their highly independent impacts on root microbiomes and plant phenotypes. However, it should be noted that this *in vitro* experiment deviated from the interactions observed in the field experiment, in which *Lotus* was grown directly at the site where the inoculated community used in this study was collected (Bamba et al. 2020). The dominance of Proteobacteria observed in this study was also observed in the field, but was even more pronounced during the growing period. In contrast, the Actinobacteria observed in the field decreased during the growing period, especially in the root microbiome. Our experimental methods explained these unique patterns. The growth pots were filled with vermiculite and media and kept anaerobic; these conditions are unfavorable for most Actinobacteria that are aerobic (Trujillo 2016). Despite these disparities, our comprehensive *in vitro* approach, and imprecise reconstruction of in natura interactions, this comprehensive approach not only helps to clarify the individual effects of these factors, but also provides valuable insight into the interactions that underpin the structure of the root microbiome and its impact on plant growth.

The majority of the *Lotus* root microbiome was determined by the sole effects of M and E, with a single effect of G, whereas the effects of their interactions remained minor. The small G effects and minor interaction effects on root microbiomes shown in our *in vitro* pot experiments were corresponding to the field experiments of Azarbad et al. (2022). In the present study, the response to E (salt treatment) differed between the soil and root microbiomes, with a larger interaction effect between M and E in the soils. The α-diversity of the soil microbiomes showed that the soil derived from the salt-treated field, F5S, contained more microbes capable of overcoming salt stress than the soil derived from F5C. This suggests that the expected interaction between M and E was observed, even with the experimental material used in this study. However, its effect on the plant root microbiome was changed, and some microbes that declined in the F5C soil microbiome under salt treatment did not decrease in the roots even under salt treatment. This suggests that the microbial sensitivity to the environment changes because of their recruitment to the soil by plants. Modification of the root microbiome in response to environmental stress by the plant has been demonstrated in a previous study (Naylor and Coleman-Derr 2017), indicating that our study potentially observed the influence of environmental factors on the root microbiome, both directly and indirectly, via the plant.

The relatively small effect of G on the root microbiome is also consistent with previously reported findings in multiple studies (Weinert et al. 2011, Bulgarelli et al. 2012, Lundberg et al. 2012, Peiffer et al. 2013, Walters et al. 2018, Brown et al. 2021). Our investigation evaluated the effect of G in the presence of M and E effects; however, even when including their interactive effects, the magnitude of G did not reach that of M and E. This may be attributed to the low abundance of microbes sensitive to G effects (such as Enterobacteriaceae (0.7%) identified in this study) within the community. Furthermore, the lack of a significant correlation between genetic distance within *Lotus* species and the similarity of root microbiomes, coupled with no clear differences in root microbiomes between *Lotus* and *L. burttii*, suggests that the genetic differences governing G effects may not be fixed among these closely related species. Typically, larger genetic dissimilarities among plants coincide with larger differences in root microbiomes (Bouffaud et al. 2014, Schlaeppi et al. 2014, Terrazas et al. 2020, Wang and Sugiyama 2020), and species with low intraspecific diversity, such as *Boechera stricta*, exhibit less noticeable G effects (Wagner et al. 2016). Considering that *Lotus* and *L. burttii* are not highly differentiated, it is suggested that only genetic variations underlying G effects on certain microbes, such as Enterobacteriaceae, have accumulated, indicating that the contribution of the *Lotus* genotype to the community structure remains relatively small.

*Lotus* growth may be more sensitive to plant–microbe interactions depending on the G than the differences among the encountered M. The smaller effect of M on plant phenotypes, particularly plant shoot phenotypes, than that of G × M indicated that the majority of the altered microbes had little effect on plant phenotypes; however, their interaction with G had more significant outcomes. These G × M effects differed between plant shoot and root phenotypes; the root phenotype was more sensitive to differences in the encountered microbiome than shoot phenotypes. This suggests that plant roots interact directly with soil microbes and respond to them in a genotype-dependent manner. In contrast, the interaction effects can be buffered/facilitated by each host genotype and spread to the shoots.

How can the G × M effects be generated? There are two possible pathways through which a particular microbe can exert its G-dependent effects. The first is when the effect of microbes on the plant is constant, but their frequency of localization to the roots depends on the plant genotype. In the present study, a few significant genotype-dependent microbes were detected, and if they are effective on plants, this pathway is likely to follow. Secondly, the effect of microbes on plants depends on the plant genotype, regardless of the frequency of the microbes. For instance, nodule bacteria are likely to follow this latter pathway because their effects are genotype-dependent (Bamba et al. 2020) (even though the effects of nodule bacteria were excluded in this study). The G × M effect is exhibited if the effects produced by these pathways are M-dependent. There are two possible scenarios for this process: either microbes showing genotype-dependent effects are distributed differently in each soil microbiome or, there was no difference in the distribution of microbes among different soil microbiomes, but genotype-dependent effects were observed in a particular microbiome, such as microbe–microbe interactions in the community. Even though around 68% of bacterial OTUs were distributed in different inoculant communities and could support the former scenario, it is still challenging to determine which scenario each G × M-related strain would follow.

It was also challenging to evaluate the effect of each bacterium on plant phenotypes because G, M, and E and their interactions affected both plant growth and the root microbiome and did not allow us to separate them. However, the most notable difference in plant growth in this study was observed as a negative effect in the inoculated groups compared to the noninoculated groups. This was not consistent across all plant accessions; a positive effect on plant growth was also detected (specifically in MG11, MG63, and MG67 under salt stress). Consequently, by extracting microbial communities from the soil and evaluating the effects of M, it is possible to identify advantageous interactions for plant growth. The ability to evaluate such effects in noninoculated groups is also one of the advantages of conducting experiments in an *in vitro* system such as the one used in this study. Accordingly, more detailed experiments and analyses, such as inoculation studies using synthetic communities (Finkel et al. 2017), would be more efficient in clarifying which microbes can affect plant phenotypes. Furthermore, by focusing on the natural diversity of *L. japonicus* (Shah et al. 2020), we can elucidate the genetic loci underlying the effects of G, G × M, and G × M × E on plant phenotypes and root microbiomes. This approach would be valuable for disentangling the shape and maintenance of plant–microbiome interactions in nature.

## Supplementary Material

fiae056_Supplemental_Files
