# Supplementary figures and images for: Synergistic effects of plant genotype and soil microbiome on growth in Lotus japonicus
Source: FEMS Microbiol Ecol. 2024 Apr 27;100(5):fiae056. doi: 10.1093/femsec/fiae056 (PMC11068475; doi:10.1093/femsec/fiae056)

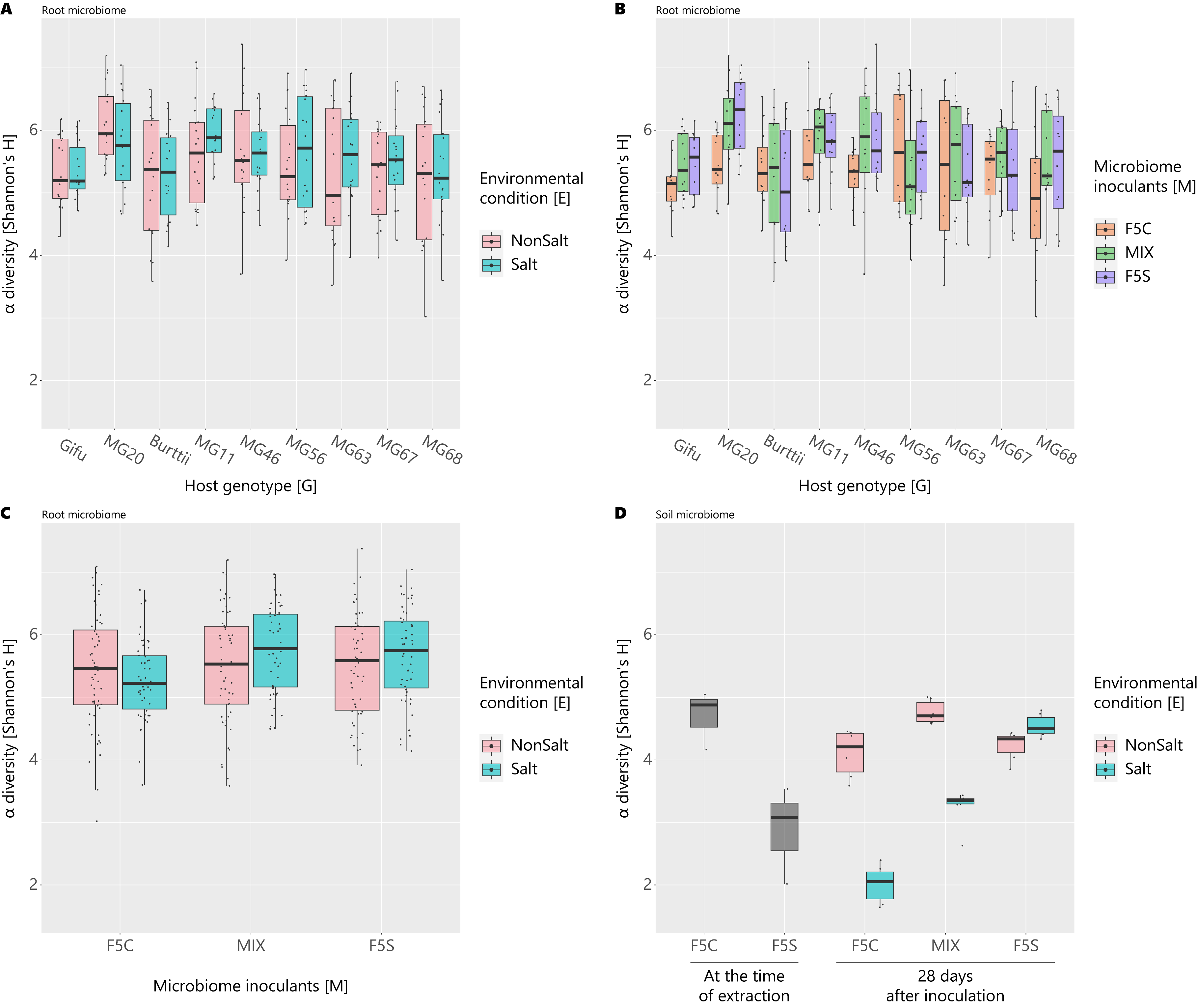

Supplement: fiae056_Supplemental_Files [file fiae056_supplemental_files.zip › Figures/Bamba_et_al_20231113_FEMS_Figure_1.tif]

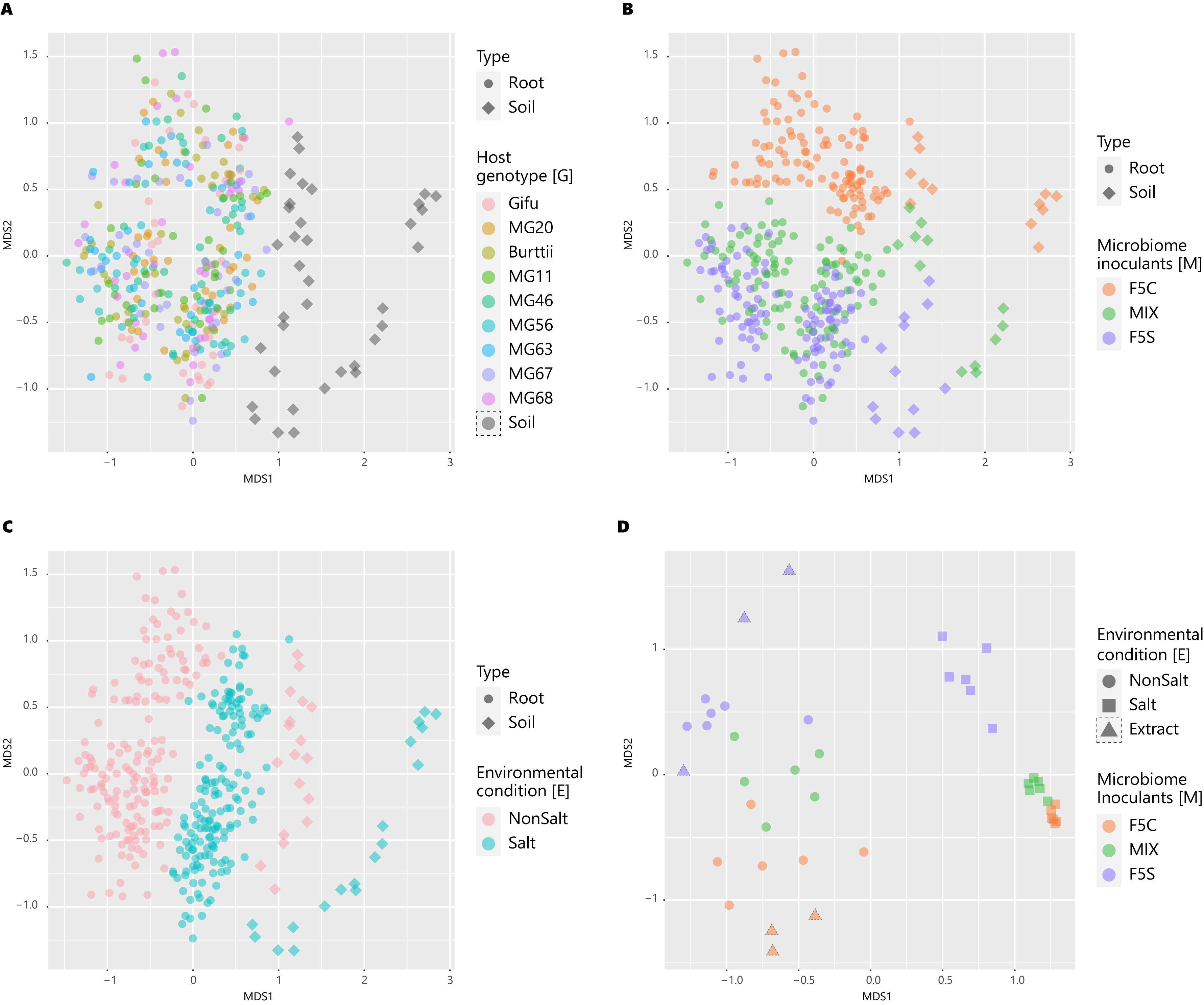

Supplement: fiae056_Supplemental_Files [file fiae056_supplemental_files.zip › Figures/Bamba_et_al_20231113_FEMS_Figure_2.tif]

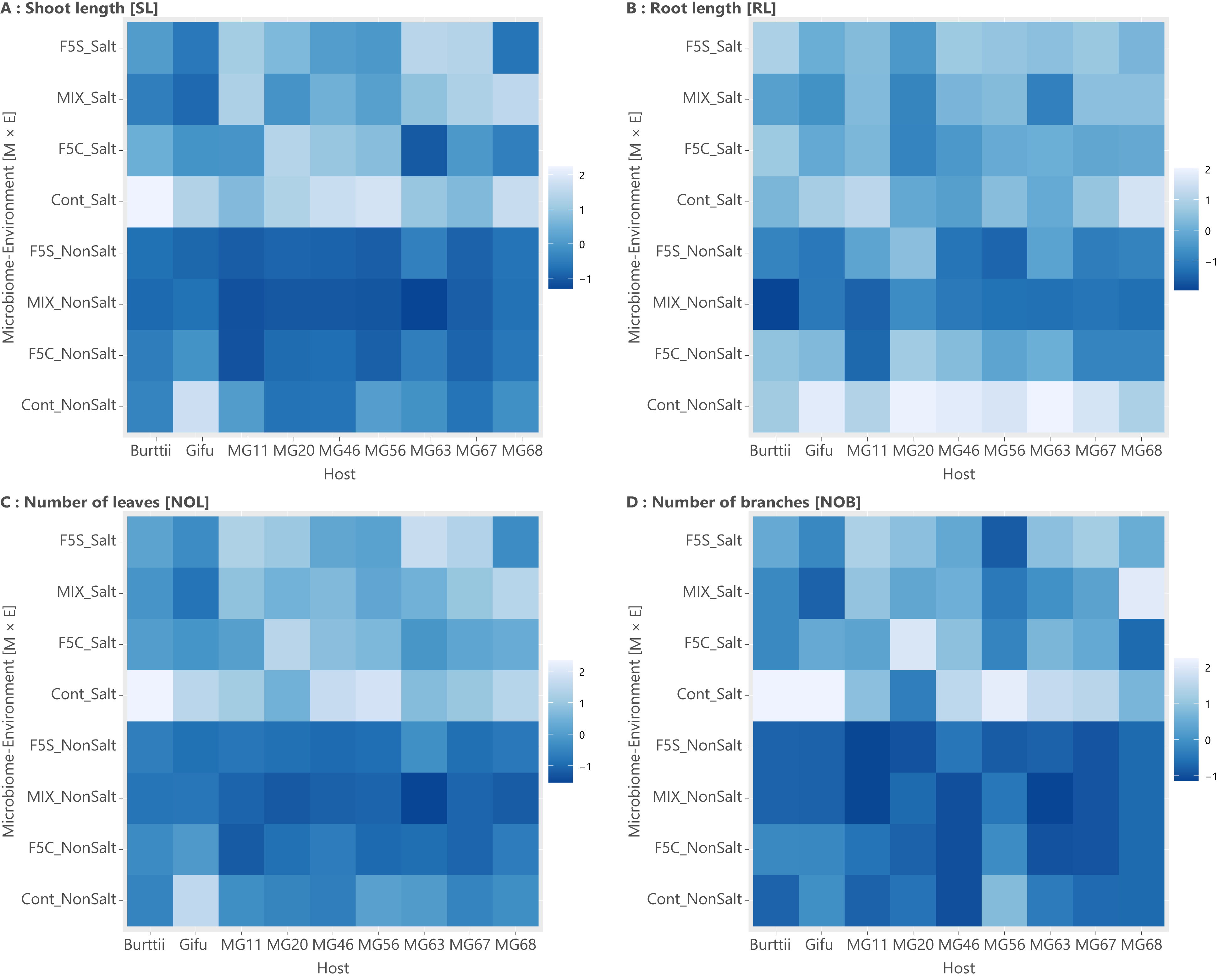

Supplement: fiae056_Supplemental_Files [file fiae056_supplemental_files.zip › Figures/Bamba_et_al_20231113_FEMS_Figure_3.tif]

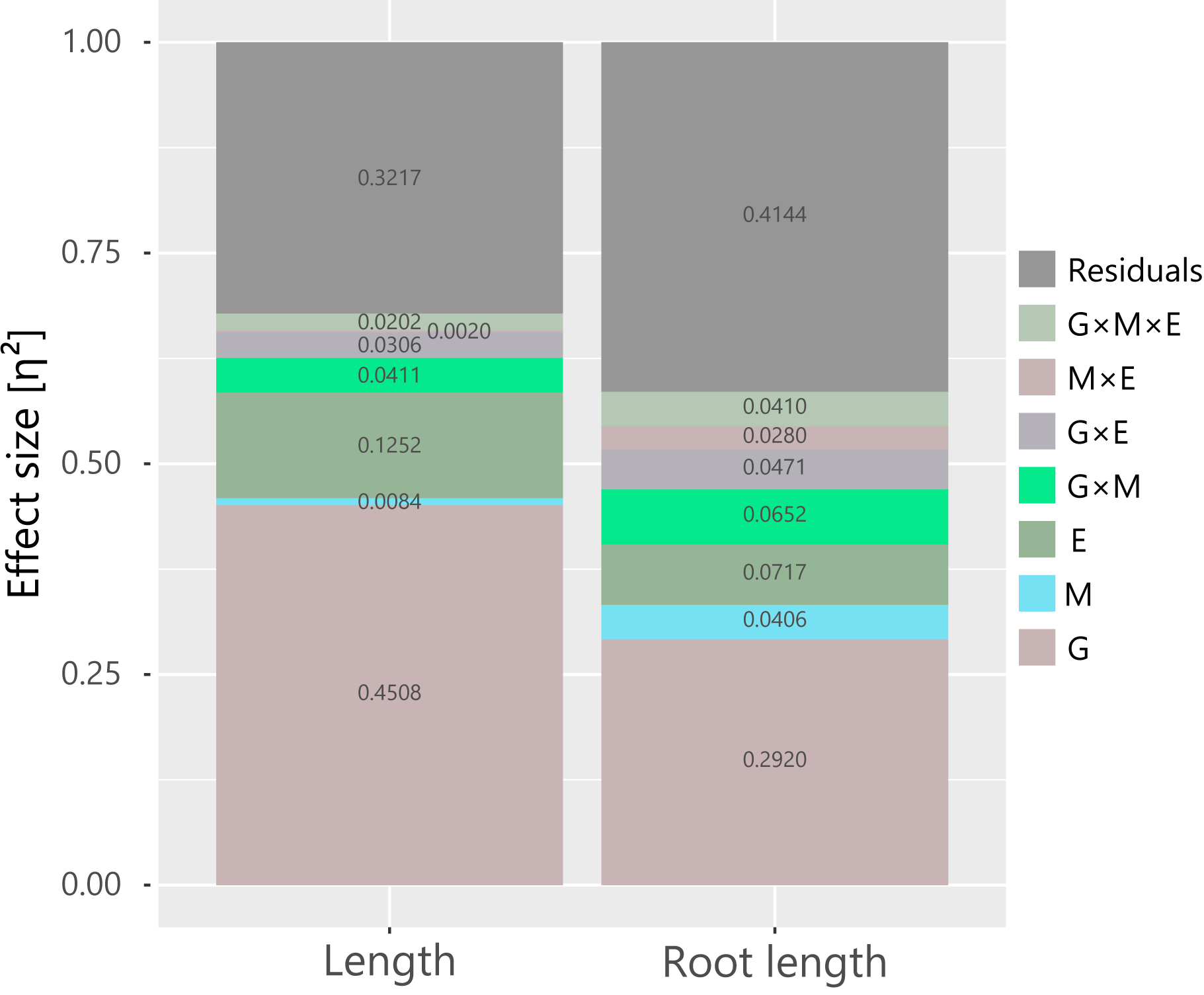

Supplement: fiae056_Supplemental_Files [file fiae056_supplemental_files.zip › Figures/Bamba_et_al_20231113_FEMS_Figure_4.tif]

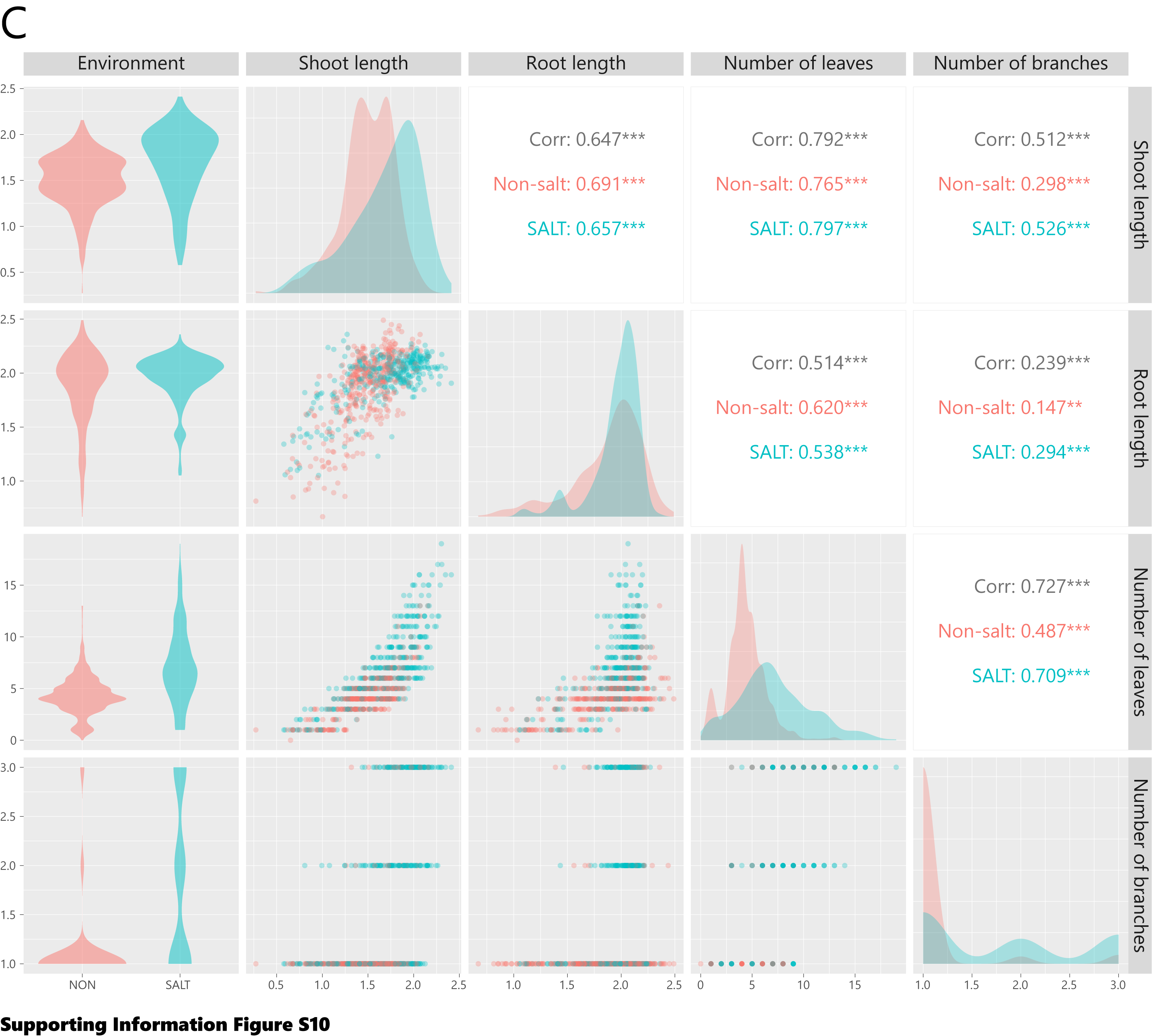

Supplement: fiae056_Supplemental_Files [file fiae056_supplemental_files.zip › Figures/Bamba_et_al_20231113_FEMS_Figure_S10_Supplementary_Data.tif]

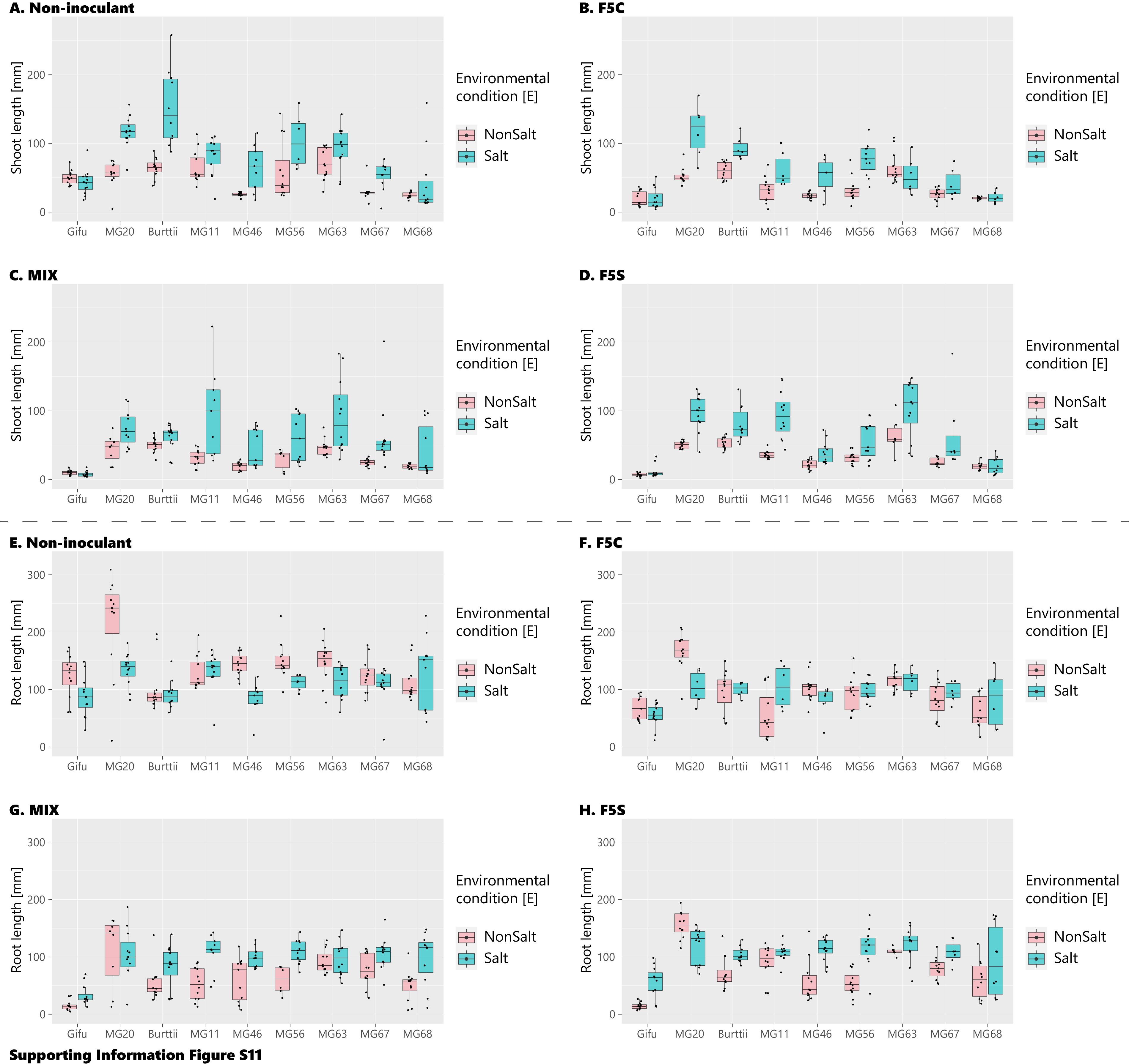

Supplement: fiae056_Supplemental_Files [file fiae056_supplemental_files.zip › Figures/Bamba_et_al_20231113_FEMS_Figure_S11_Supplementary_Data.tif]

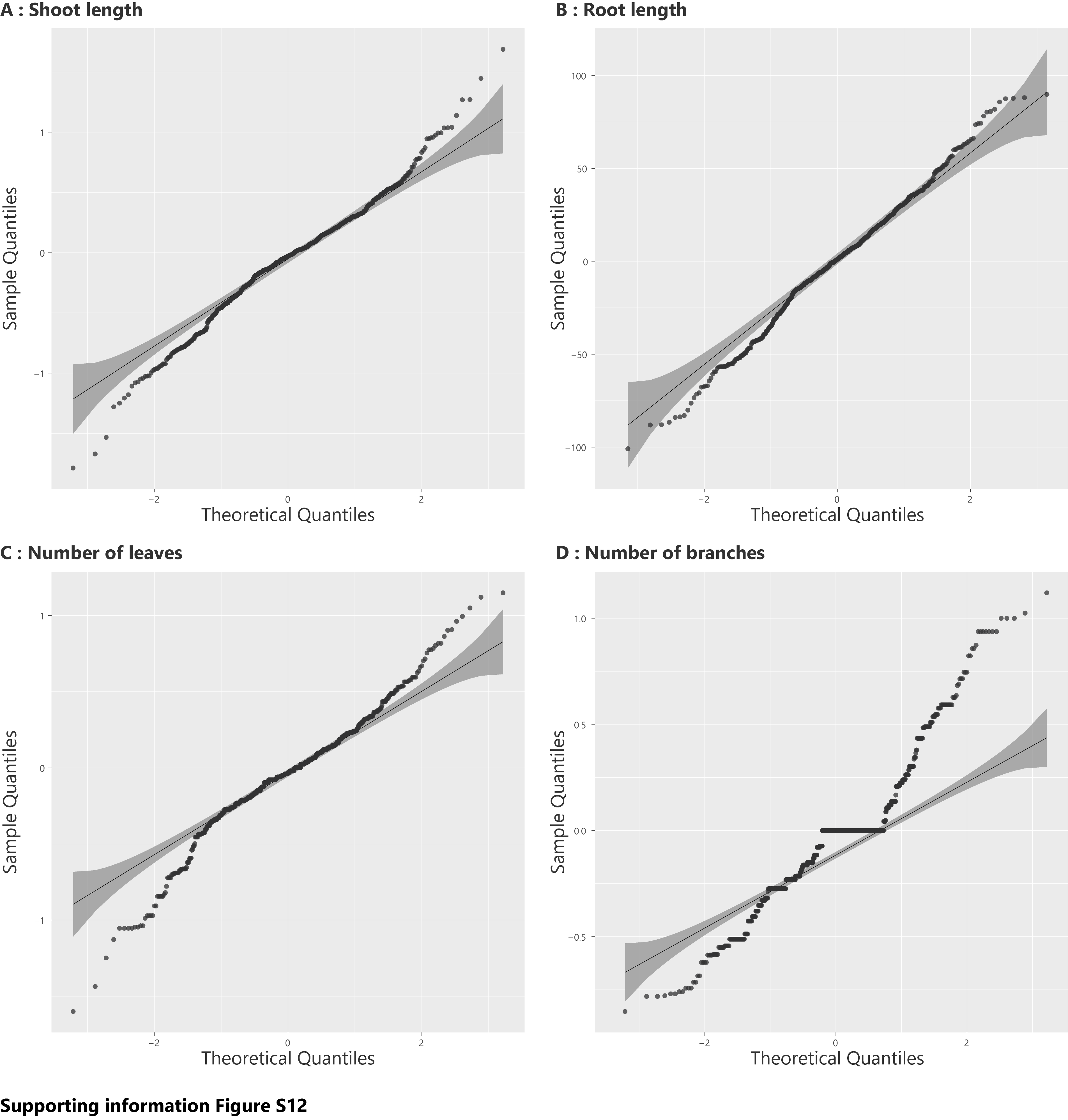

Supplement: fiae056_Supplemental_Files [file fiae056_supplemental_files.zip › Figures/Bamba_et_al_20231113_FEMS_Figure_S12_Supplementary_Data.tif]

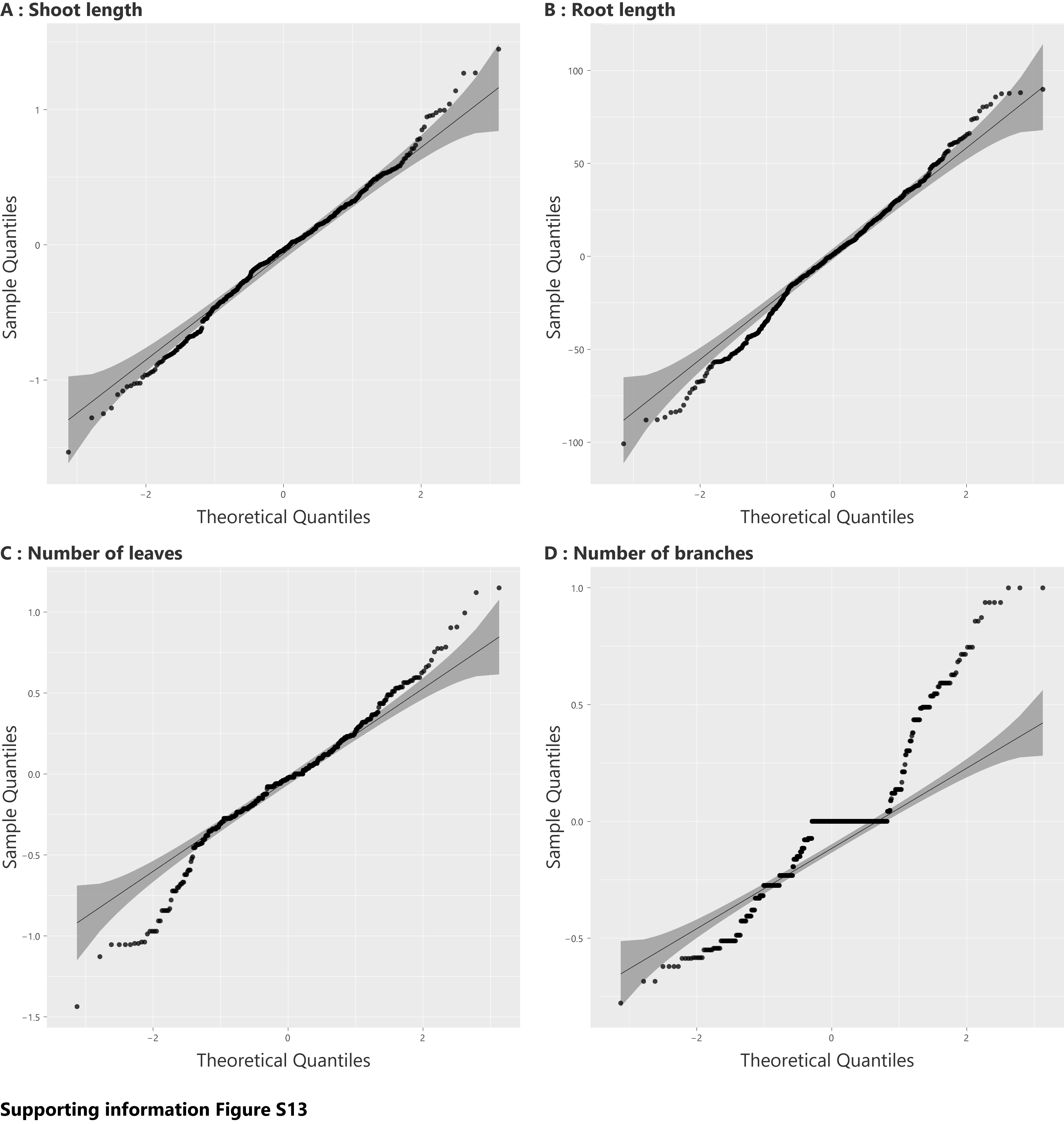

Supplement: fiae056_Supplemental_Files [file fiae056_supplemental_files.zip › Figures/Bamba_et_al_20231113_FEMS_Figure_S13_Supplementary_Data.tif]

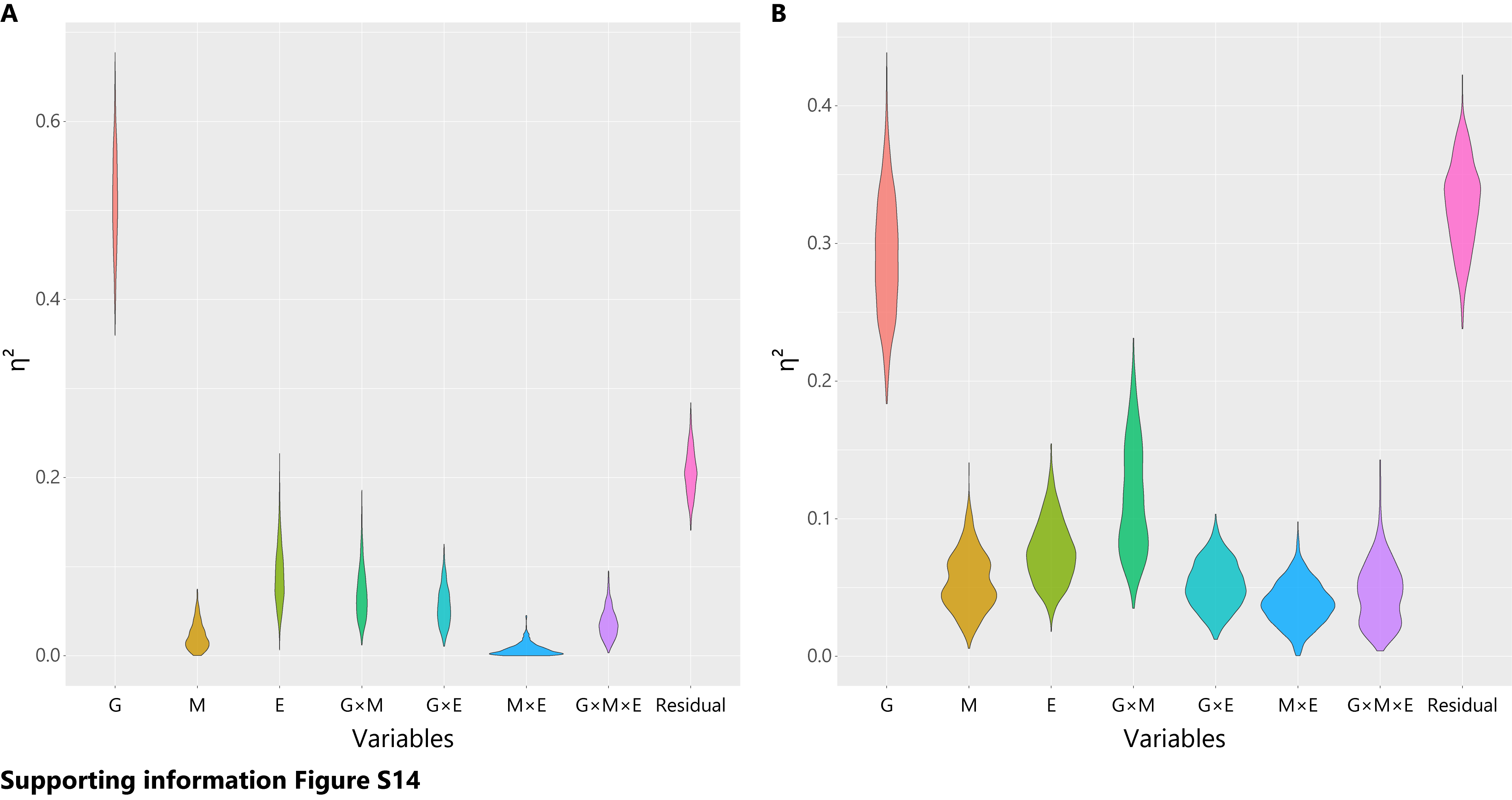

Supplement: fiae056_Supplemental_Files [file fiae056_supplemental_files.zip › Figures/Bamba_et_al_20231113_FEMS_Figure_S14_Supplementary_Data.tif]

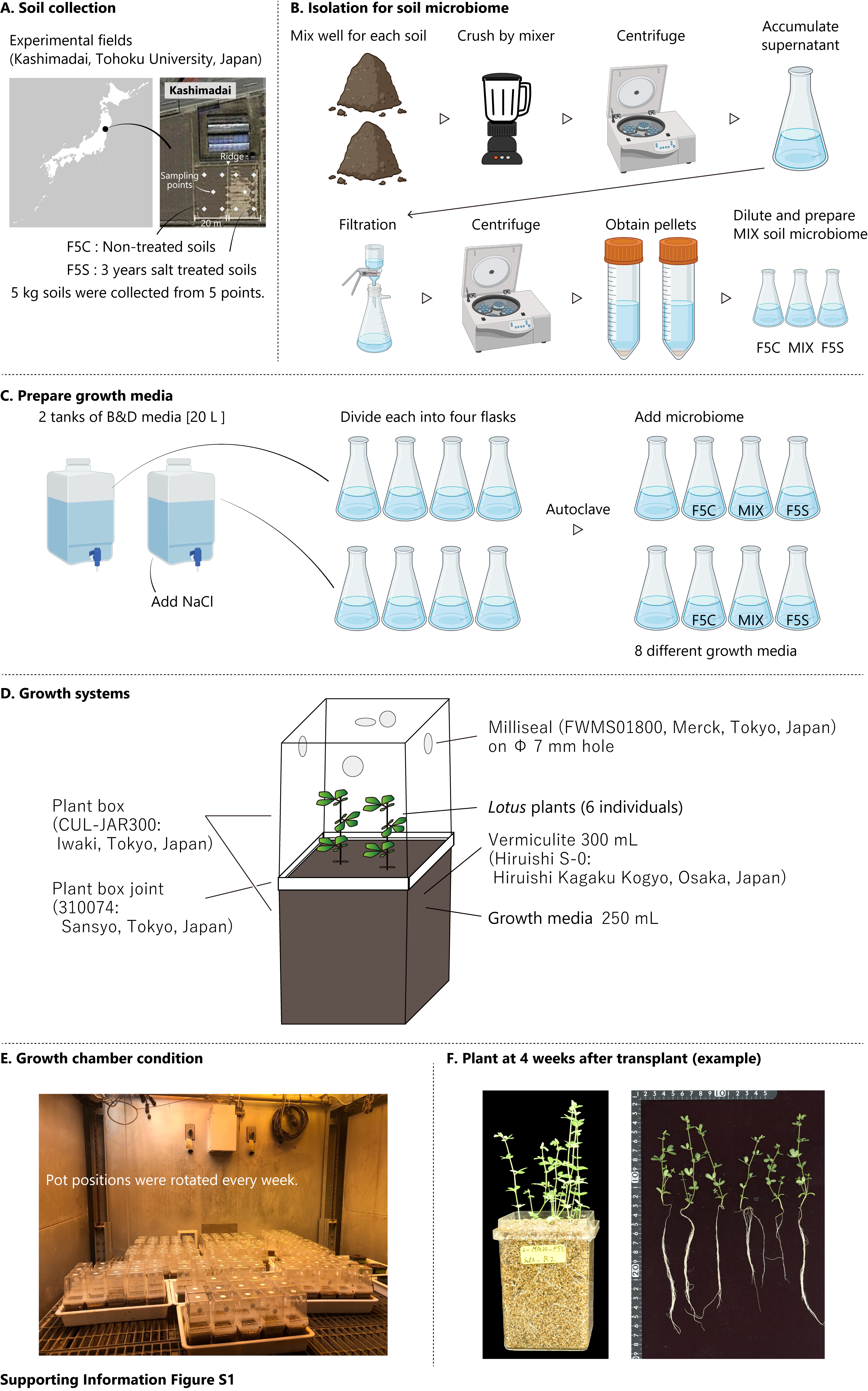

Supplement: fiae056_Supplemental_Files [file fiae056_supplemental_files.zip › Figures/Bamba_et_al_20231113_FEMS_Figure_S1_Supplementary_Data.tif]

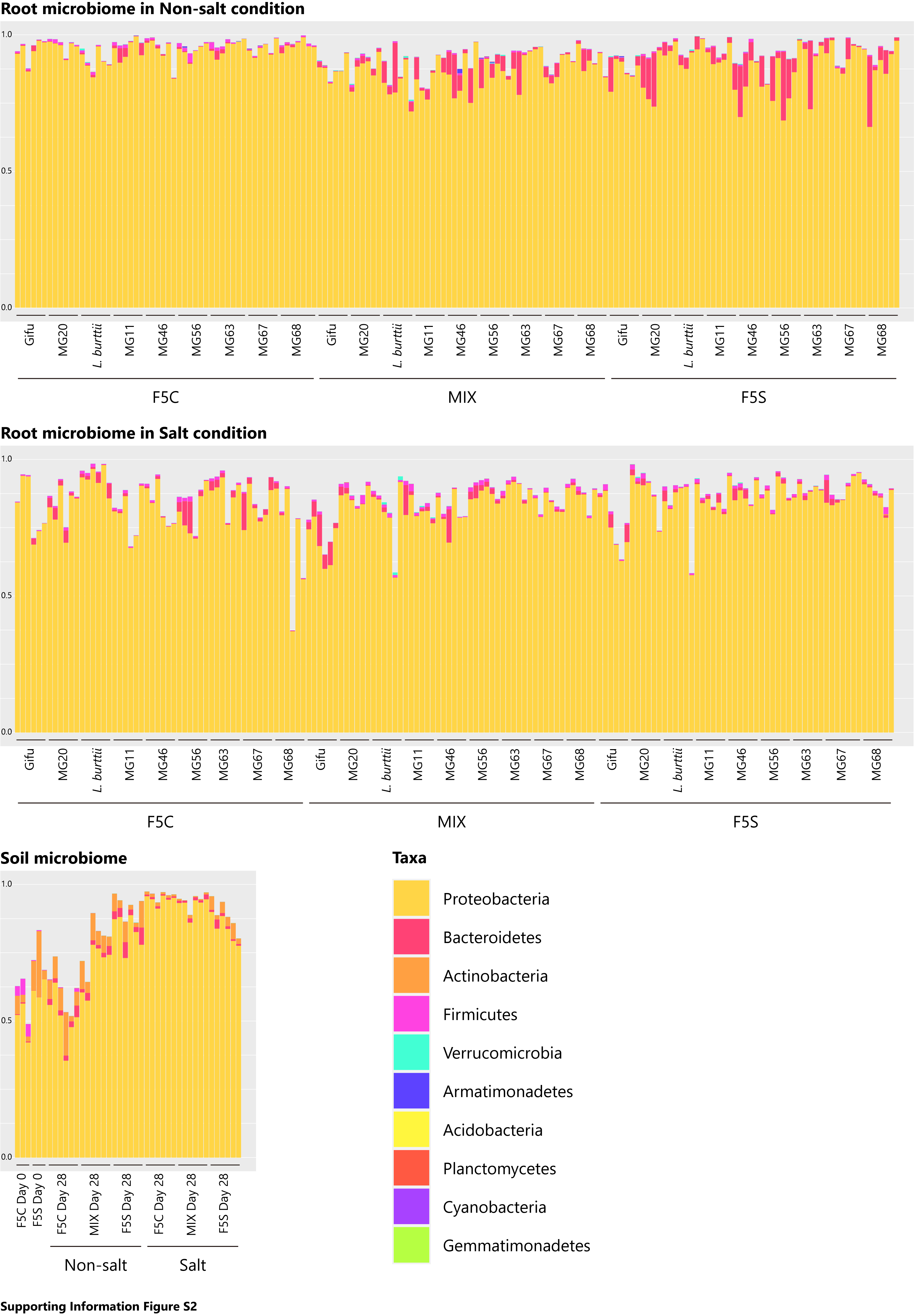

Supplement: fiae056_Supplemental_Files [file fiae056_supplemental_files.zip › Figures/Bamba_et_al_20231113_FEMS_Figure_S2_Supplementary_Data.tif]

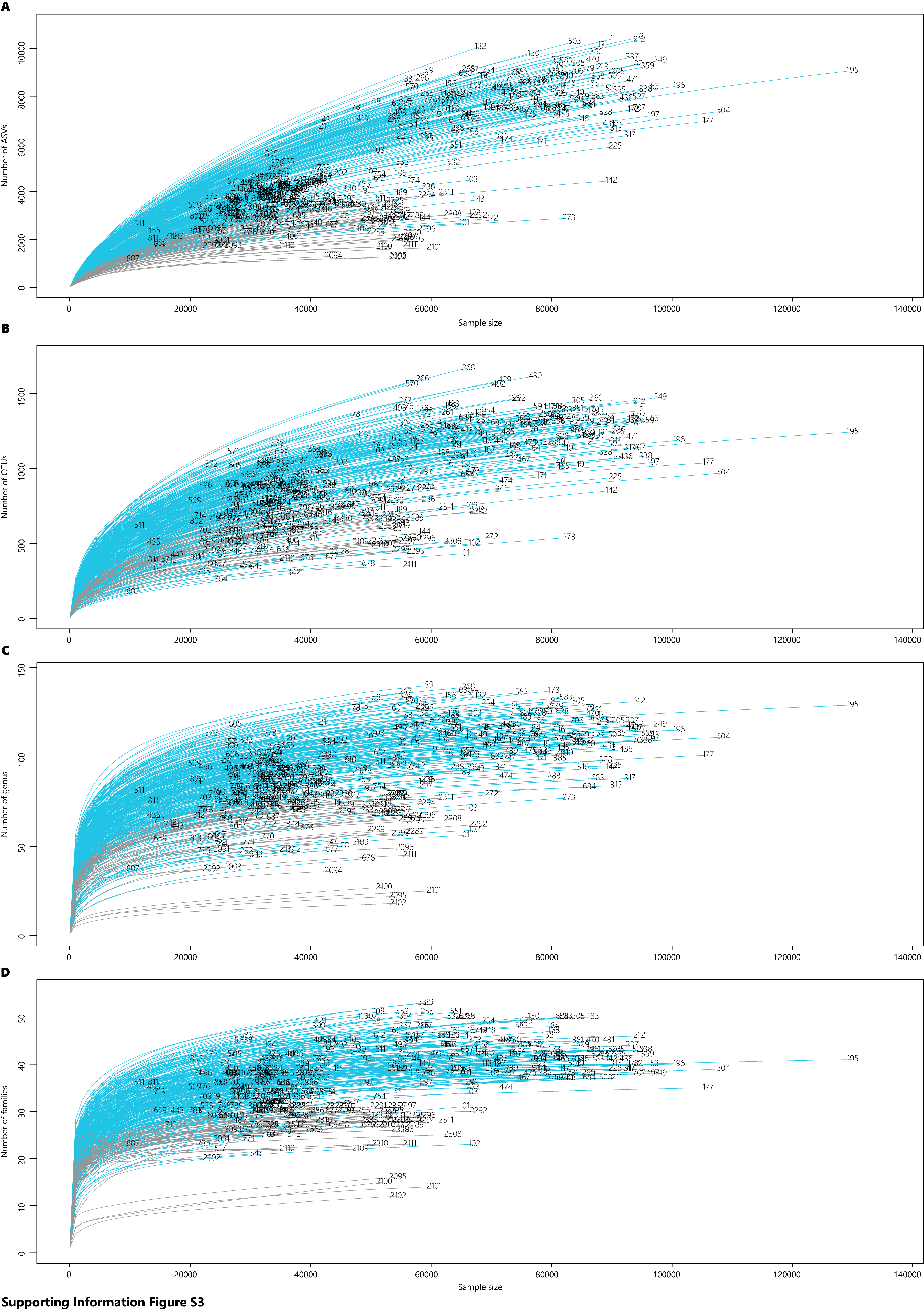

Supplement: fiae056_Supplemental_Files [file fiae056_supplemental_files.zip › Figures/Bamba_et_al_20231113_FEMS_Figure_S3_Supplementary_Data.tif]

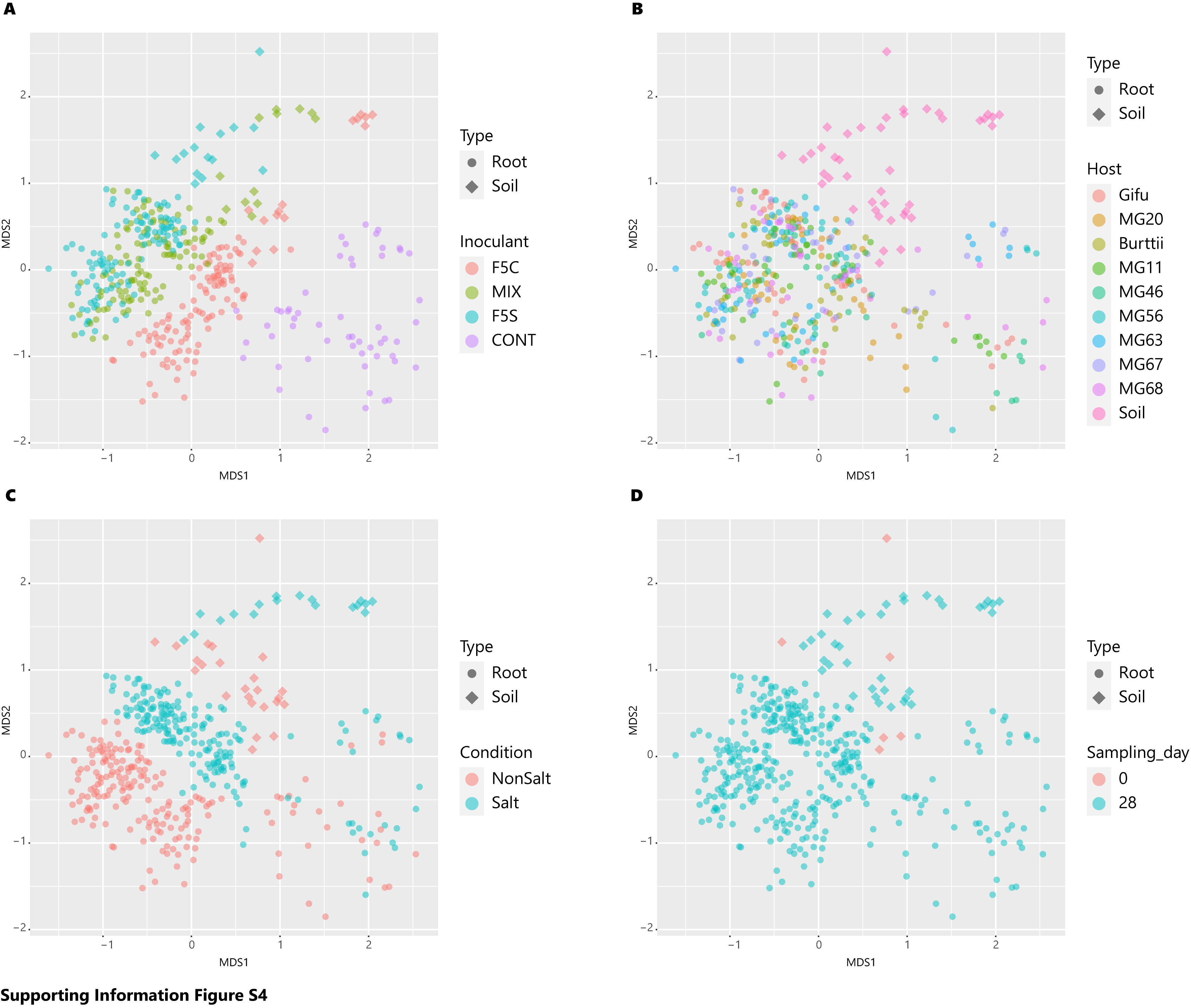

Supplement: fiae056_Supplemental_Files [file fiae056_supplemental_files.zip › Figures/Bamba_et_al_20231113_FEMS_Figure_S4_Supplementary_Data.tif]

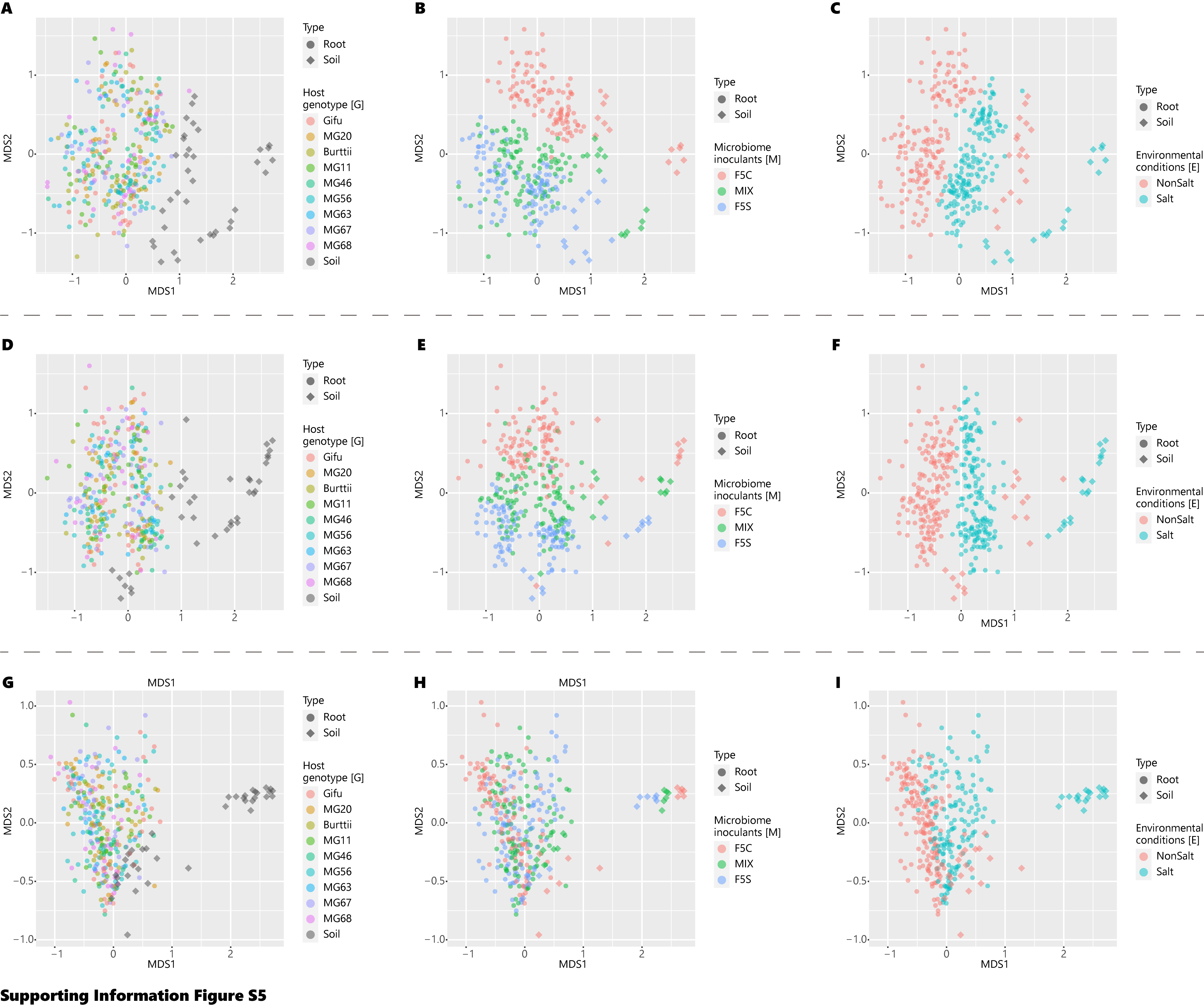

Supplement: fiae056_Supplemental_Files [file fiae056_supplemental_files.zip › Figures/Bamba_et_al_20231113_FEMS_Figure_S5_Supplementary_Data.tif]

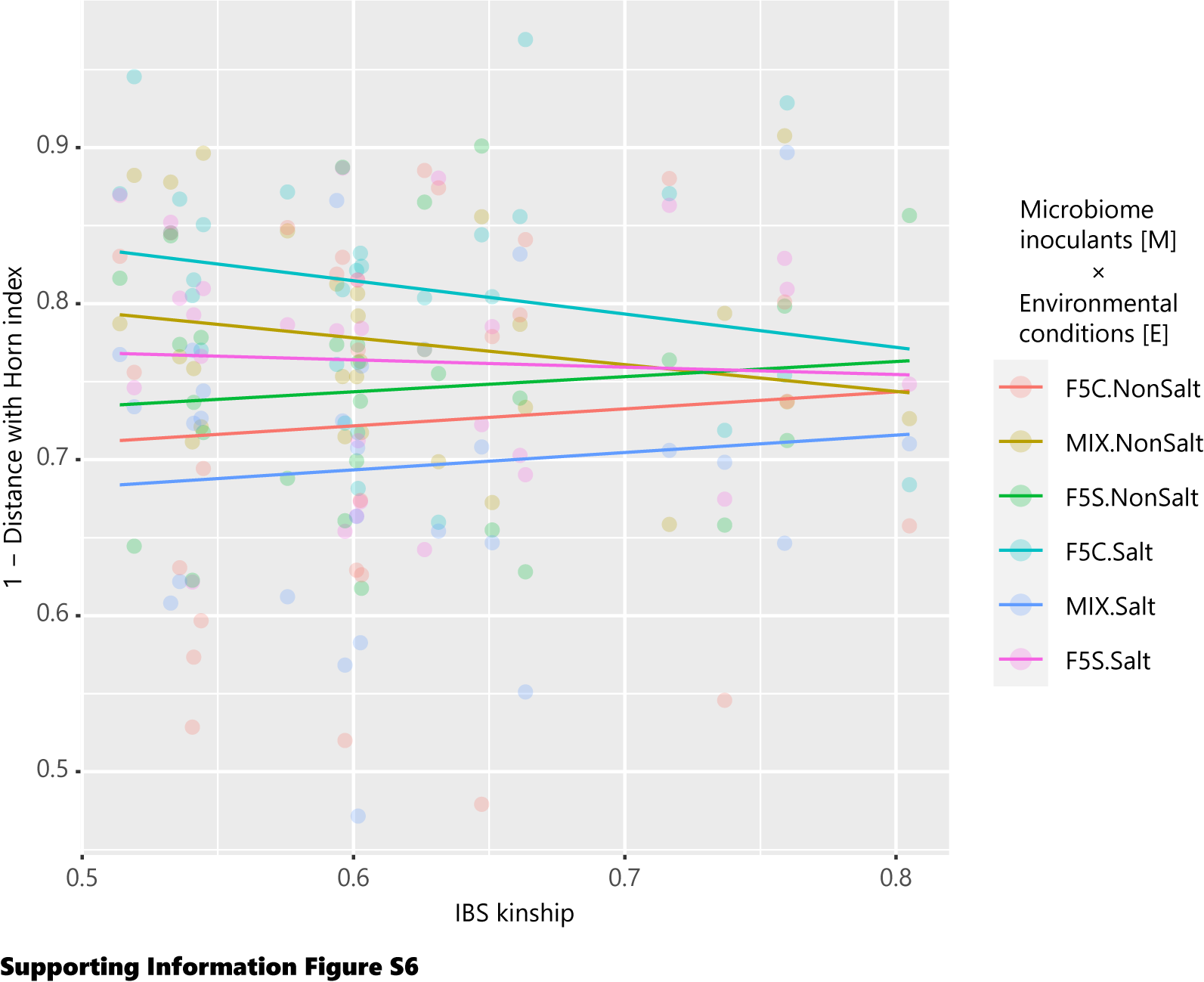

Supplement: fiae056_Supplemental_Files [file fiae056_supplemental_files.zip › Figures/Bamba_et_al_20231113_FEMS_Figure_S6_Supplementary_Data.tif]

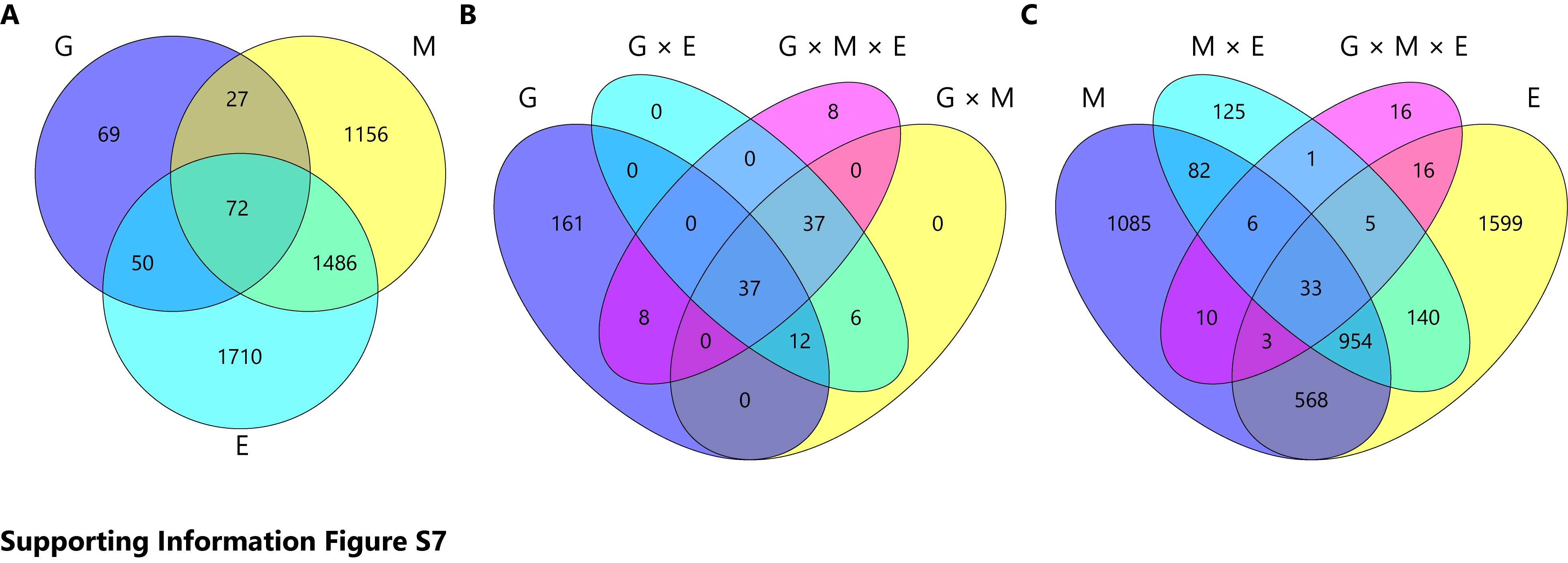

Supplement: fiae056_Supplemental_Files [file fiae056_supplemental_files.zip › Figures/Bamba_et_al_20231113_FEMS_Figure_S7_Supplementary_Data.tif]

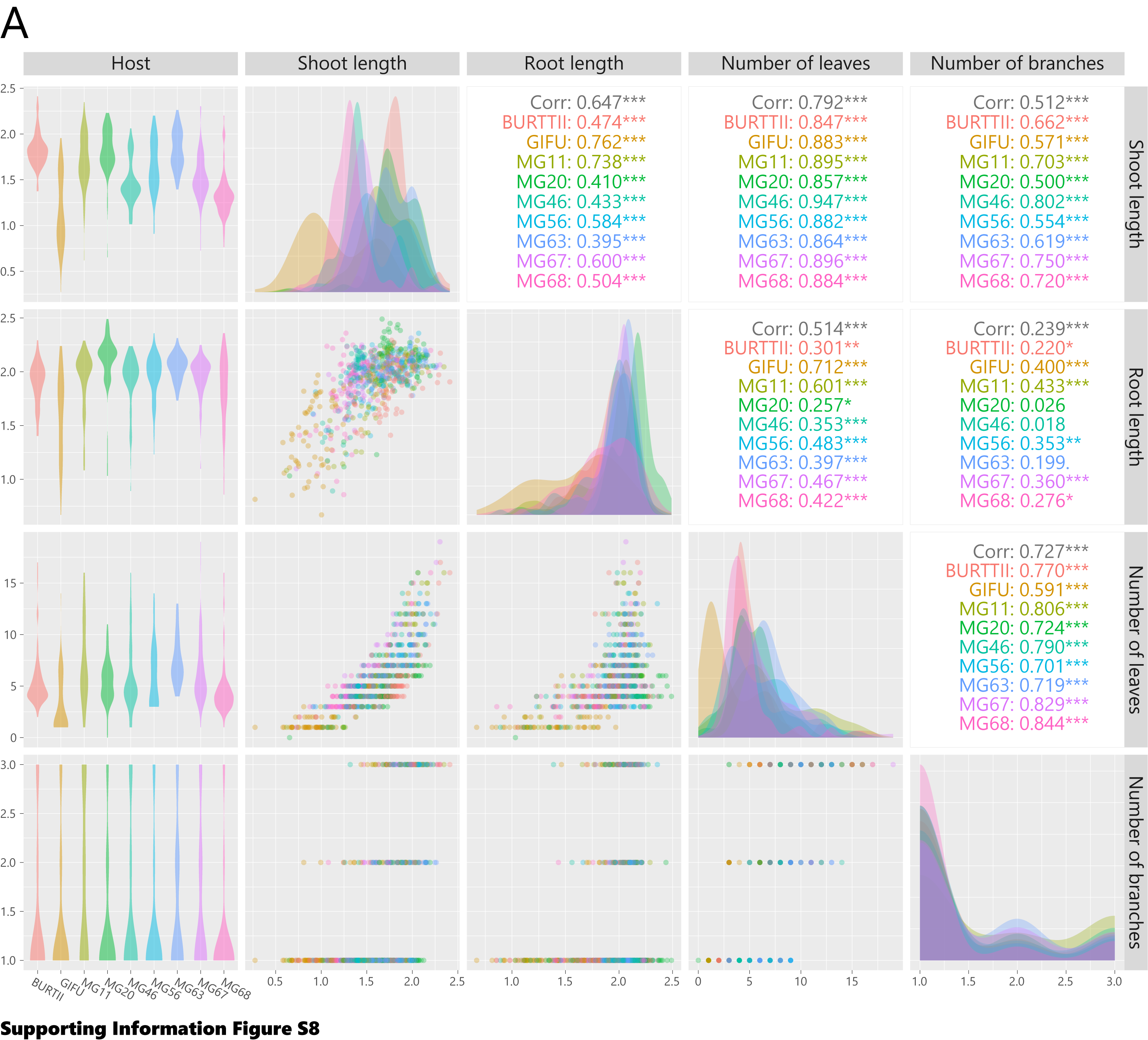

Supplement: fiae056_Supplemental_Files [file fiae056_supplemental_files.zip › Figures/Bamba_et_al_20231113_FEMS_Figure_S8_Supplementary_Data.tif]

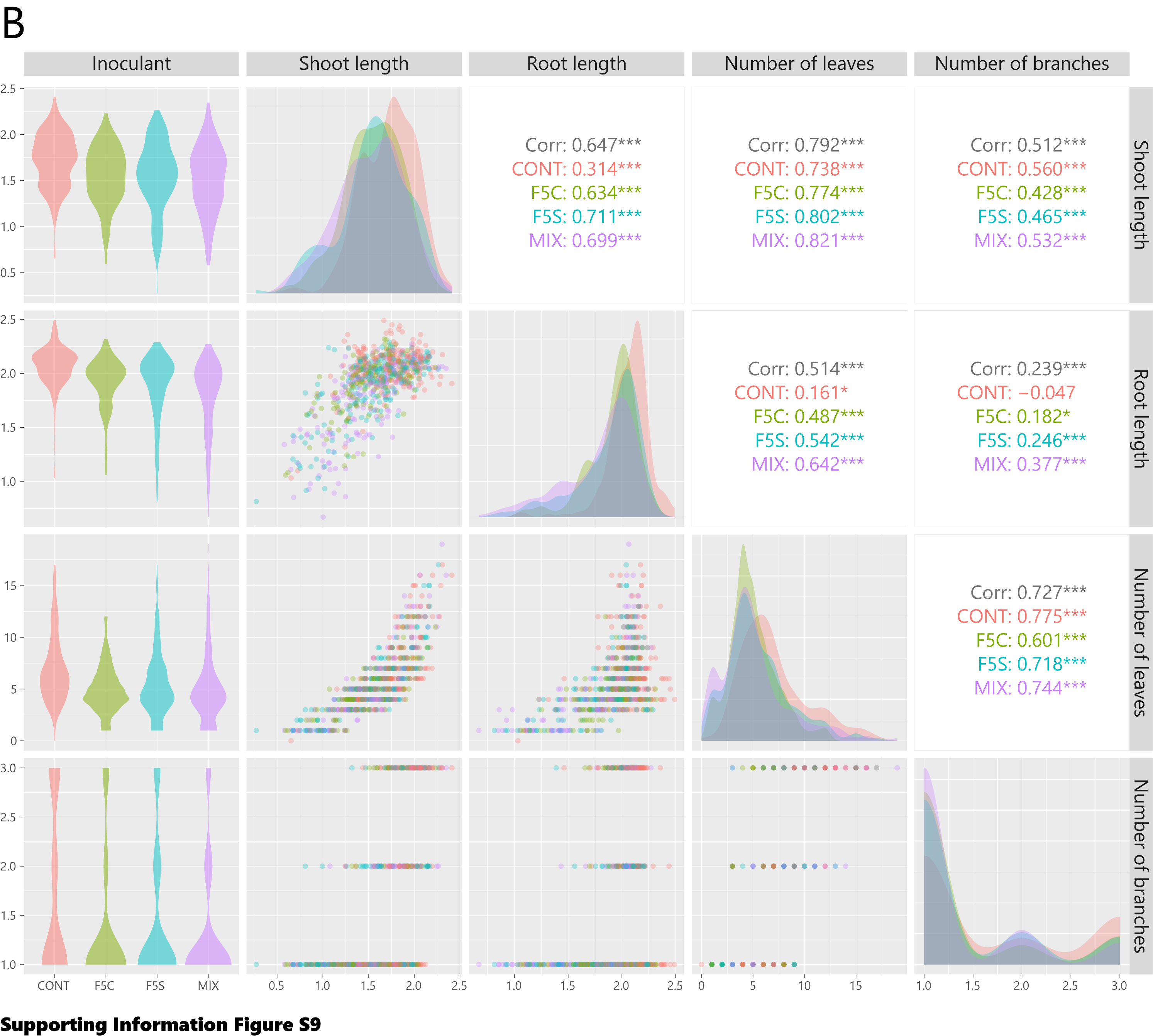

Supplement: fiae056_Supplemental_Files [file fiae056_supplemental_files.zip › Figures/Bamba_et_al_20231113_FEMS_Figure_S9_Supplementary_Data.tif]
